# Integrated analysis of microRNA and mRNA expression profiles in *Crassostrea gigas* to reveal functional miRNA and miRNA-targets regulating shell pigmentation

**DOI:** 10.1038/s41598-020-77181-0

**Published:** 2020-11-19

**Authors:** Dandan Feng, Qi Li, Hong Yu, Shikai Liu, Lingfeng Kong, Shaojun Du

**Affiliations:** 1 Key Laboratory of Mariculture, Ministry of Education, Ocean University of China, Qingdao, 266003 China; 2 Laboratory for Marine Fisheries Science and Food Production Processes, Qingdao National Laboratory for Marine Science and Technology, Qingdao, 266237 China; 3 Department of Biochemistry and Molecular Biology, Institute of Marine and Environmental Technology, University of Maryland School of Medicine, Baltimore, MD USA

**Keywords:** Computational biology and bioinformatics, Molecular biology

## Abstract

MicroRNAs (miRNAs) regulate post-transcription gene expression by targeting genes and play crucial roles in diverse biological processes involving body color formation. However, miRNAs and miRNA-targets underlying shell color polymorphism remain largely unknown in mollusca. Using four shell colors full-sib families of the Pacific oyster *Crassostrea gigas*, we systematically identified miRNAs and miRNA-targets in the mantles, which organ could produce white, golden, black or partially pigmented shell. RNA sequencing and analysis identified a total of 53 known miRNA and 91 novel miRNAs, 47 of which were detected to differentially express among six pairwise groups. By integrating miRNA and mRNA expression profiles, a total of 870 genes were predicted as targets of differentially expressed miRNAs, mainly involving in biomineralization and pigmentation through functional enrichment. Furthermore, a total of four miRNAs and their target mRNAs were predicted to involve in synthesis of melanin, carotenoid or tetrapyrrole. Of them, lgi-miR-317 and its targets *peroxidase* and lncRNA TCONS_00951105 are implicated in acting as the competing endogenous RNA to regulate melanogenesis. Our studies revealed the systematic characterization of miRNAs profiles expressed in oyster mantle, which might facilitate understanding the intricate molecular regulation of shell color polymorphism and provide new insights into breeding research in oyster.

## Introduction

The large proportion of a eukaryotic genome is transcribed to produce a huge array of RNA molecules differing in protein-coding capability, size, and abundance^1^. Non-coding RNAs (ncRNAs) genes produce functional RNA molecules, which comprised of different types of small noncoding RNAs (sRNAs), long noncoding RNAs (lncRNAs) implicated in transcriptional and post-transcriptional regulation of gene expression or in guiding DNA modification^2,3^. The most well-studied ncRNAs are microRNAs (miRNAs)^4^, a class of endogenous sRNAs, which regulate post-transcription gene expression by targeting protein-coding mRNAs or other ncRNAs for cleavage or translational repression^5^. Furthermore, each miRNA may repress up to hundreds of transcripts^6^, and mediate competing endogenous RNAs (ceRNAs) network, which regulate other RNA transcripts by competing for shared miRNAs^7^.

miRNAs have been shown to play crucial roles in diverse biological processes, including development^8^, immune response^9^, body color formation^10^ and so on. Emerging evidence indicated that miRNAs may have important roles in pigmentation. For example, the small RNA libraries analysis of pigmented and non-pigmented skin suggests a functional relevance of miRNA in the modulation of pigmentation processes in alpaca^11^, goat^12^ and fish^10,13^. In mollusca, the fabulous and diverse shell colors are generally believed to be determined by presence of biological pigments of melanin, carotenoid and tetrapyrrole^14^. At present, the studies on molecular mechanism related to the pigmentation processes have mainly focused on mRNAs, little information is available on the roles of miRNAs in shell pigmentation^15^.

The Pacific oyster, *Crassostrea gigas*, is a widely distributed mariculture shellfish species, ranking first in production among all aquatic animals in the world^16^. Through successive family selection and breeding, four shell color strains of *C. gigas* (white, WS; golden, GS; black, BS and partially pigmented, NS) have been developed to improve the commercial values. A better understand on the molecular mechanism of shell pigmentation will facilitate the genetic breeding of shell color variants. Digital gene expression profiling (DGE) and lncRNAs transcriptome analysis discovered some differentially expressed (DE) genes and enriched pathways potentially involved in pigmentation, using those four shell color full-sib families^17,18^. In *C. gigas*, miRNAs from different tissues and developmental stages have recently been reported, involving development^19^, immune^9^ and osmotic stress response^20^. However, the miRNAs profiles in mantle of four shell color variants have not been characterized, and their association with shell pigmentation remains to be discovered.

In this study, we investigated miRNAs profiles in mantle of *C. gigas* characterized by shell color (WS, GS, BS, NS) using high-throughput sequencing. Based on the released genome sequence of *C. gigas*^21^, the known and novel miRNAs were identified from mantle of four shell colors variants. By integrating with DE mRNAs assemblies and lncRNAs profiles from same four shell color oyster families, the target mRNAs and lncRNAs of DE miRNAs were identified and the ceRNA networks were constructed. The miRNAs and their targets related to shell pigmentation were identified and analyzed. This comprehensive database of miRNAs could provide a valuable resource for studying function of miRNAs in mantle of *C. gigas*, as well as contributes to better understanding the molecular mechanism of shell pigmentation.

## Materials and methods

### Sample collection and preparation

Four kinds of *C. gigas* lines of full-sib families (WS, BS, GS, NS) were established, which were developed by seven-generation successive family selection and exhibited stably heritable shell color traits^18^. The original parents of white, black, golden and normal *C. gigas* were selected from locally cultured populations in Weihai, Shandong, China^18^. In each full-sib family, six oyster individuals were sampled for RNA-seq. Left mantle was dissected and immediately stored by liquid nitrogen for RNA extraction.

### Small RNA library construction and sequencing

The total RNA was extracted from mantle of each individual using Trizol Reagent (Invitrogen, Carlsbad, CA) according to the manufacturer’s instructions. Approximately 3 µg of total qualified RNA was pooled proportionally from six individuals within each family, a total of four samples were used as input material for the small RNA library^18^. Sequencing libraries were generated using NEBNext Multiplex Small RNA Library Prep Set for Illumina (NEB, USA) following manufacturer’s instructions. Library quality was assessed on the Agilent Bioanalyzer 2100 system using DNA High Sensitivity Chips. The prepared libraries were sequenced on an Illumina Hiseq 2500 platform at Novogene (Tianjing, China) and 50 bp single-end reads were generated.

### Data analysis and quality control

The complete dataset of raw data was deposited into NCBI`s Sequence Read Archive under the accession number PRJNA628161. Clean reads were obtained by removing reads containing ploy-N, with 5′ adapter contaminants, without 3′ adaptor or the insert tag, containing ploy A/T/G/C and low quality reads from raw data^22^. Then, clean reads of 18–35 bp length were chosen to do all the downstream analyses.

### Identification of miRNAs

The total sRNA tags were mapped to the oyster genome databases by Bowtie^23^ without mismatch to analyze their expression and distribution on the reference. Mapped small RNA tags were used to look for known miRNA from miRBase 21.0 (ftp://mirbase.org/pub/mirbase/). Modified software mirdeep2^24^ and srna-tools-cli (https://srna-tools.cmp.uea.ac.uk) were used to obtain the potential miRNA. All clean reads were mapped to RepeatMasker, Rfam database to remove reads originating from protein-coding genes, repeat sequences, rRNA, small nuclear RNA (snRNA), and small nucleolar RNA (snoRNA). The novel miRNAs were predicted using software miREvo^25^ and mirdeep2^26^. To make every unique small RNA mapped to only one annotation, we summarized all alignments and annotations following the priority rule: known miRNA > rRNA > snRNA > snoRNA > repeat > gene > novel miRNA.

### Quantification of miRNA

The expression levels of miRNAs were estimated by TPM (the tag counts per million aligned miRNAs)^27^. Differential expression analysis of two samples was performed using the DEGseq R package^28^. *P*-value was adjusted using q*-*value^29^. The threshold for significantly differential expression were *q-*value < 0.01 and |log_2_(foldchange)| > 1.

### Target gene prediction for miRNAs in oyster mantle

Predicting the target gene of miRNA was performed by miRanda^30^. To get more precise miRNA–mRNA correlation information, the target genes of DE miRNAs were integrated with the DE genes found by the corresponding pair-wise comparison^18^. Since most miRNAs were found to repress mRNA translation or induce mRNA degradation, the datasets in our study were analyzed through intersecting elements of target genes of (1) DE miRNAs and DE mRNAs, (2) down-regulated miRNAs and up-regulated mRNAs and (3) up-regulated miRNAs and down-regulated mRNAs. GOSeq software^31^ and KOBAS software^32^ were used to annotate the functions of target genes and perform the statistical enrichment analysis.

### Construction the lncRNA–miRNA–mRNA networks

The lncRNA database sequenced from the same four families was used to predict miRNAs targets by miRanda^30^. Before prediction, the lncRNAs were filtered based the homology. In addition, the miRNA–lncRNA pairs were selected by opposite expression regulation. Then the predicted miRNA–lncRNA pairs were integrated with the miRNA–mRNA pairs by the shared miRNA. The lncRNA–miRNA–mRNA interaction networks of DE-miRNAs and their corresponding targets were constructed using Cytoscape (https://www.cytoscape.org/). And we focused on six previously identified pigmentation related genes^18^, which were verified by quantitative real-time PCR (qRT-PCR). The pigmentation related lncRNA–miRNA–mRNA interaction networks were selected and analyzed.

### Validation of miRNA expression by quantitative PCR analysis

Eight miRNAs were selected for qRT-PCR using gene-specific primers and universal reverse primers and U6 was used as the internal control^33^ (Supplementary Table S1). The stem-loop primer was used for miRNA reverse transcription; qRT-PCR forward primer and universal reverse primer were used to amplify miRNA sequences. Reverse transcription of miRNA was carried out using the cDNA Synthesis Kit (TaKaRa, China). The qRT-PCR experiments were conducted on Roche Lightcycler 480 (Roche, USA). The reactions were carried out in a total volume of 20 μl containing 2 μl of diluted cDNA, 0.8 μl of each primer, 0.4 μl ROX Reference Dye (50×) (TaKaRa, China), 6 μl sterile distilled water and 10 μl SYBR Premix Ex Taq II (TaKaRa, China) with the following cycling profile: 95 ℃ for 3 min, followed by 45 cycles at 95 ℃ for 15 s, 60 ℃ for 30 s. Three independent biological replicates were performed. All of the measurements were made in triplicate. The relative expression levels were calculated using 2^−△△Ct^ method.

## Results

### Analysis and identification of small RNA

A total of 57,748,768 raw reads were obtained from four libraries (BS, GS, WS and NS mantle small RNA libraries) by deep sequencing. After filtering out the low quality reads based on base quality value and removing adaptor sequences, 56,576,919 (97.97%) clean reads were retrieved, corresponding to 3,630,904 unique sequences (Table 1). A total of 47,664,086 (84.25%) reads were matched to the oyster genome in four libraries. The representations of different types of sRNAs in four libraries are shown (Table 1). The total rRNA proportion was only 1.7% (996,313/56,576,919), indicating high quality of the sample.

**Table 1:** Statistics of small RNA sequences of the four libraries.

Group of reads	Number of sequences				
	BS	WS	GS	NS	Total
Raw reads	14,760,788	14,500,420	14,022,802	14,464,758	57,748,768
Clean reads	14,550,079	14,193,934	13,724,802	14,108,104	56,576,919
Unique reads	1,019,668	1,043,540	849,230	718,466	3,630,904
Mapped total reads	11,784,143	11,230,529	11,927,774	12,721,640	47,664,086
**Known_miRNA**					
Mapped mature	52	52	52	53	53
Mapped uniq sRNA	592	611	603	554	2,360
Mapped total sRNA	3,236,054	2,893,039	3,783,154	4,805,845	14,718,092
rRNA	308,630	205,310	203,707	278,666	996,313
tRNA	1	1	0	2	4
snRNA	1373	1272	951	1072	4668
snoRNA	427	617	427	261	1732
Repeat	337,186	331,764	282,938	245,884	1,197,772
Exon	631,704	624,603	727,351	1,270,818	3,254,476
Intron	1,251,534	1,057,509	1,129,958	1,399,182	4,838,183
**novel_miRNA**					
Mapped mature	75	69	73	73	91
Mapped uniq Srna	562	566	545	528	2201
Mapped total sRNA	716,384	682,840	784,954	687,530	2,871,708

*WS* white shell oysters, *GS* golden shell oysters, *BS* black shell oysters, *NS* partially pigmented shell oysters.

**Figure 1:**
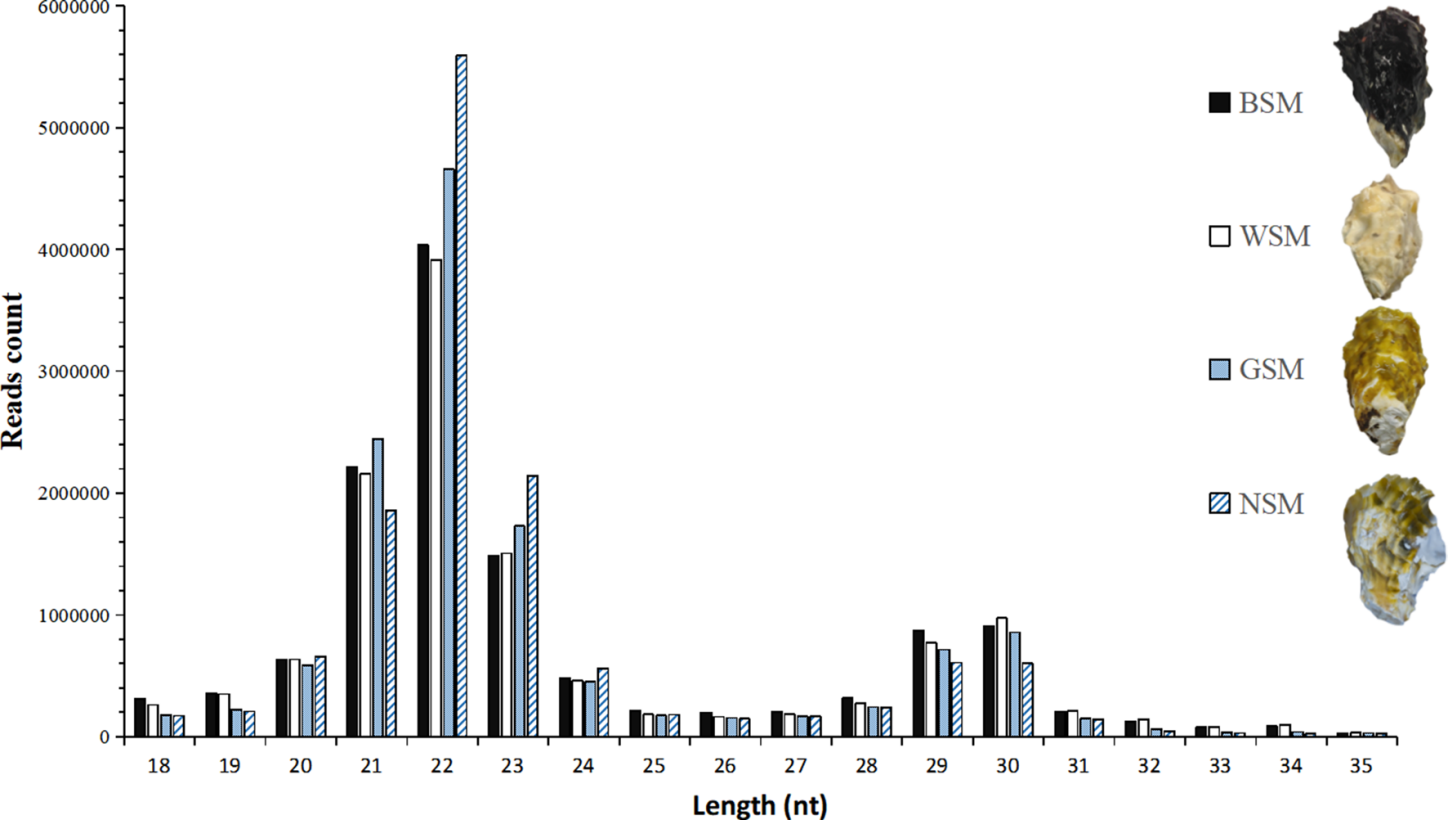
Length distribution of small RNAs in four groups from *Crassostrea gigas.* The *y*-axis represents the numbers of small RNA identified in each library, the *x*-axis represents the length of small RNA.

The length distribution of sRNAs was represented in Fig. 1, the majority of the sRNAs sequenced were 21–23 nt in length, and the most frequent length of sRNA was 22 nt. By aligning the sRNAs to all known animal miRNA precursors, 2360 (14,718,092 total reads) unique sequences were identified as potentially conserved miRNA in oyster mantle. 2201 (2,871,708 total reads) unique sequences were identified as potentially novel miRNA in oyster mantle.

After alignment and additional sequence analysis, a total of 144 candidate miRNAs from mantle libraries were identified, including 53 known miRNAs and 91 novel miRNAs (Supplementary Table S2). Approximately 38 miRNA families were represented in each library (Supplementary Table S2). All miRNA families only had 1 to 2 members, of which three families have two members, mir-1994 (lgi-mir-1994a, lgi-mir-1994b), mir-216 (lgi-mir-216a, lgi-mir-216b), mir-10 (lgi-mir-10, lgi-mir-100).

### Differential expression of miRNAs among four groups

The abundance of all the miRNAs was normalized and calculated by TPM method. In all four libraries, miRNAs with TPM ≥ 1000 were classified as abundant while those with TPM < 1 were classified as rare. 53 out of 144 miRNAs are abundant in four libraries, and 14 out of 144 miRNAs are rarely expressed (Supplementary Table S2). And lgi-miR-100, lgi-miR-1, lgi-miR-10, lgi-miR-184, lgi-miR-279, lgi-miR-8, lgi-miR-7, lgi-miR-9-5p, lgi-miR-133-3p, lgi-let-7 were the ten most abundant conserved miRNA represented in four libraries. A total of 114 miRNAs were identified to be with > 1 TPM in each library.

**Figure 2:**
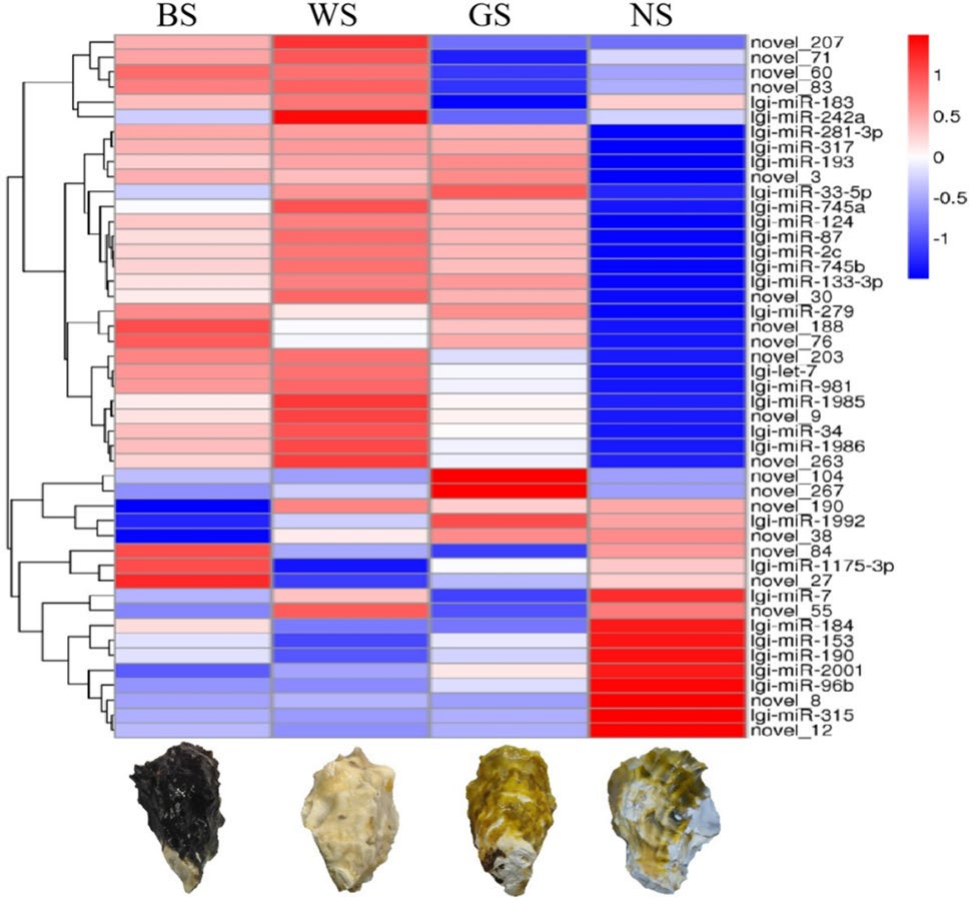
Hierarchical clustering of differentially expressed miRNAs among four shell colors variants of *Crassostrea gigas*. MiRNA with lower expression level is in blue and higher expression in red.

A total of 47 miRNAs exhibited differential expression patterns from six pair-wise comparisons. A total number of 30, 27, 26, 6, 8 and 9 DE miRNAs were respectively detected from the comparison of WS vs NS, GS vs NS, BS vs NS, BS vs WS, GS vs WS and BS vs GS. Of that, a total number of 22, 16, 16, 4, 2 and 4 miRNAs were respectively identified as up-regulated. The whole expression of DE miRNAs was shown in the Hierarchical clustering heatmap (Fig. 2). There was larger number of DE miRNAs when NS was compared with other three samples (WS, BS, GS).

### Target gene prediction for miRNAs in oyster mantle

In order to provide insight into potential biological roles of miRNAs identified in oyster mantle, target prediction analysis was performed for oyster mantle miRNAs. At first, the 47 DE miRNAs was input to miRanda software to predict the target genes, which resulted in 77,721 miRNA-target pairs. Then we selected the miRNA-target pairs, when a miRNA and its target gene were negatively correlated. A total of 870 DE genes were identified as the target gene, and there were 322, 304, 313, 126, 127 and 124 DE genes identified as putative target from WS vs NS, GS vs NS, BS vs NS, BS vs WS, GS vs WS and BS vs GS comparisons, respectively (Supplementary Table S3).

**Figure 3:**
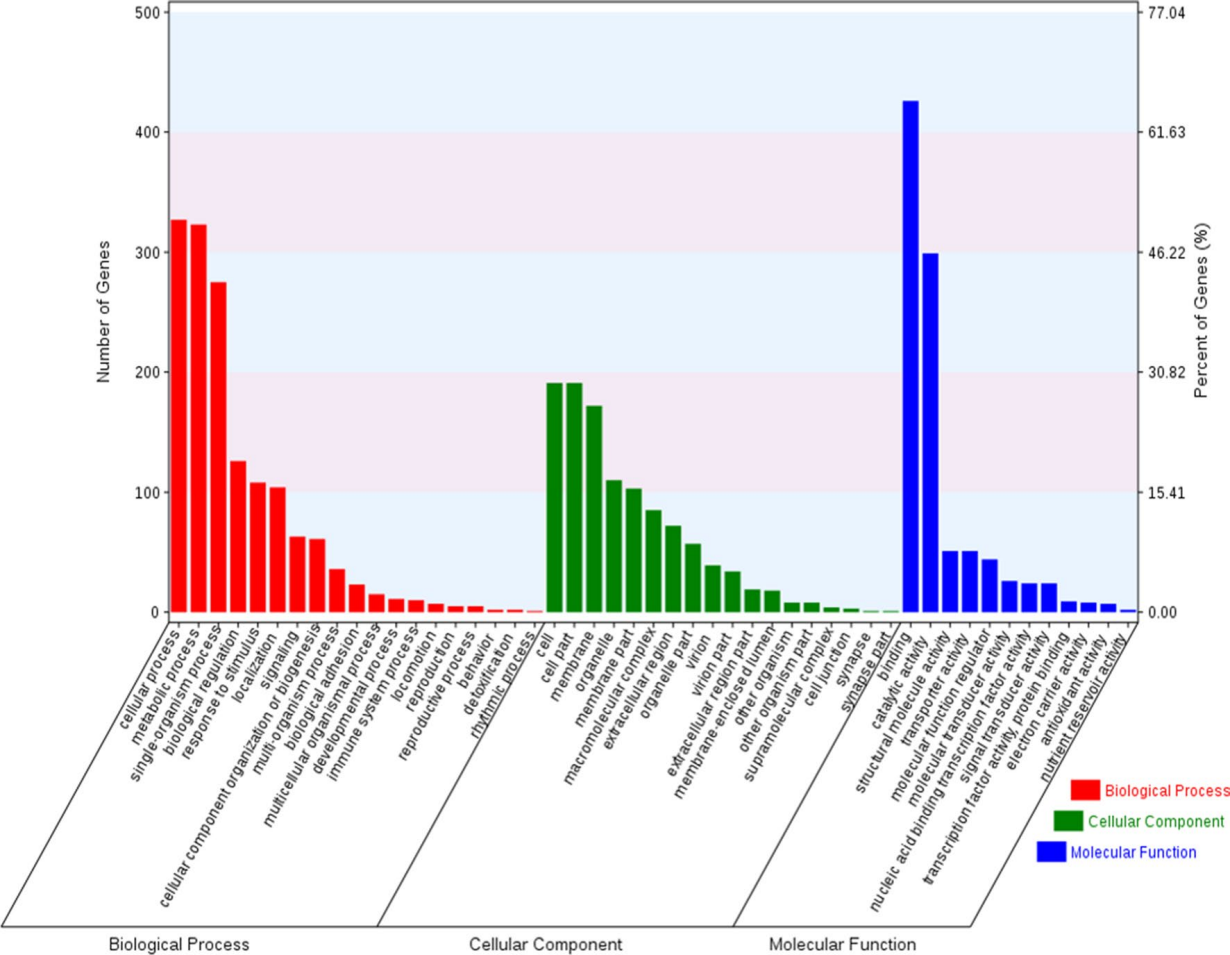
Gene ontology distribution of the target genes for all differentially expressed miRNAs among four shell colors variants of *Crassostrea gigas*.

**Figure 4:**
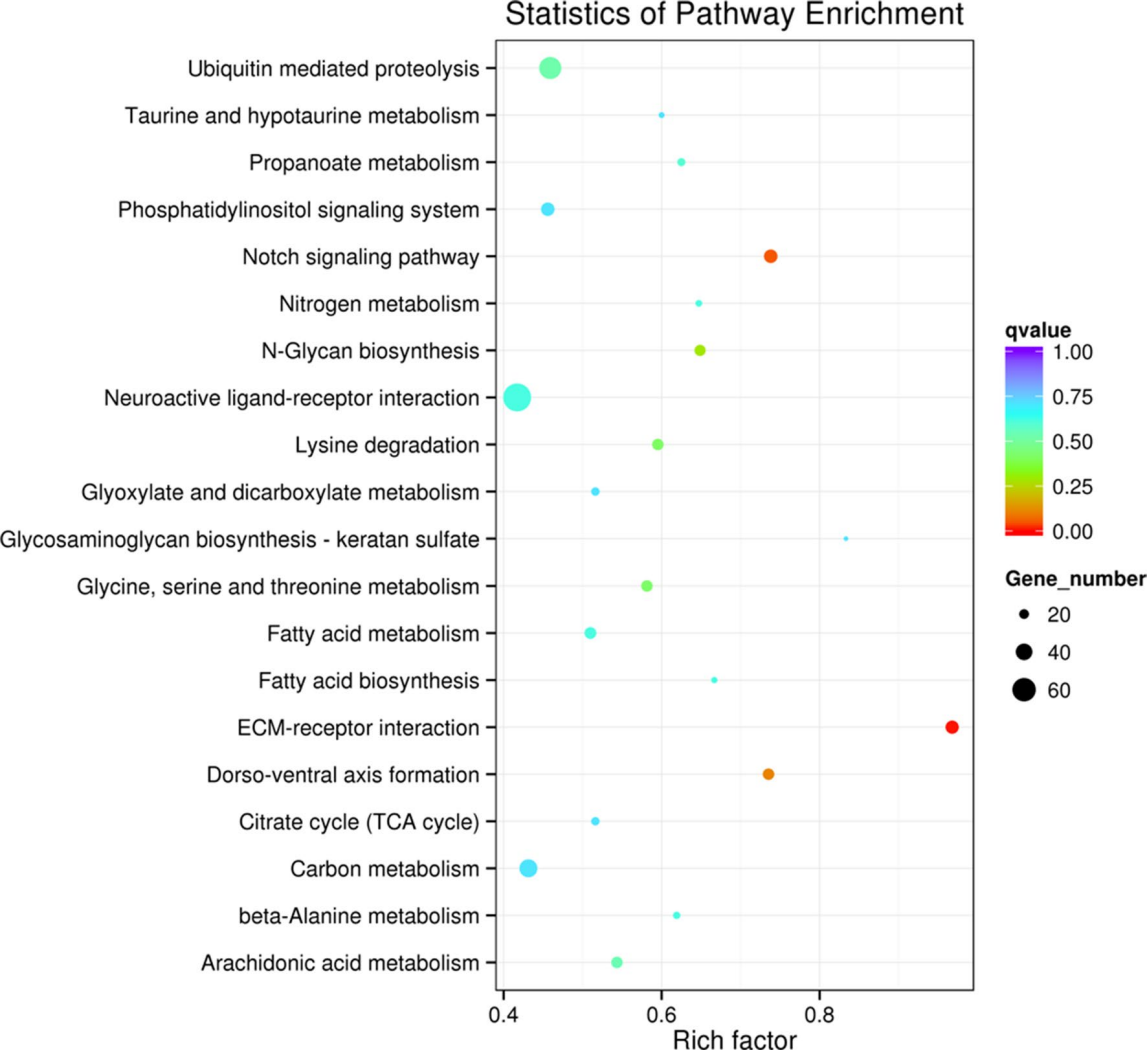
The KEGG enrichment pathways of target genes of differentially expressed micrRNAs in WS vs BS comparison. The *y*-axis represents the KEGG enriched pathways, the *x*-axis represents the enrichment factor, which was calculated by ratio of the number of differentially expressed mRNAs divided by the number of annotated mRNAs in this pathway.

The Gene ontology (GO) distribution of the predicted targets is shown (Fig. 3). In molecular function, targeted genes mainly involved in binding and catalytic activity. In biological process, targeted genes mainly related to cellular process, metabolic process and single-organism process. Only two significantly enriched KEGG pathways were detected (*P*-value < 0.05) in all six comparisons. Both ECM-receptor interaction and Notch signaling pathway were significantly enriched in WS vs NS comparison (Fig. 4).

**Figure 5:**
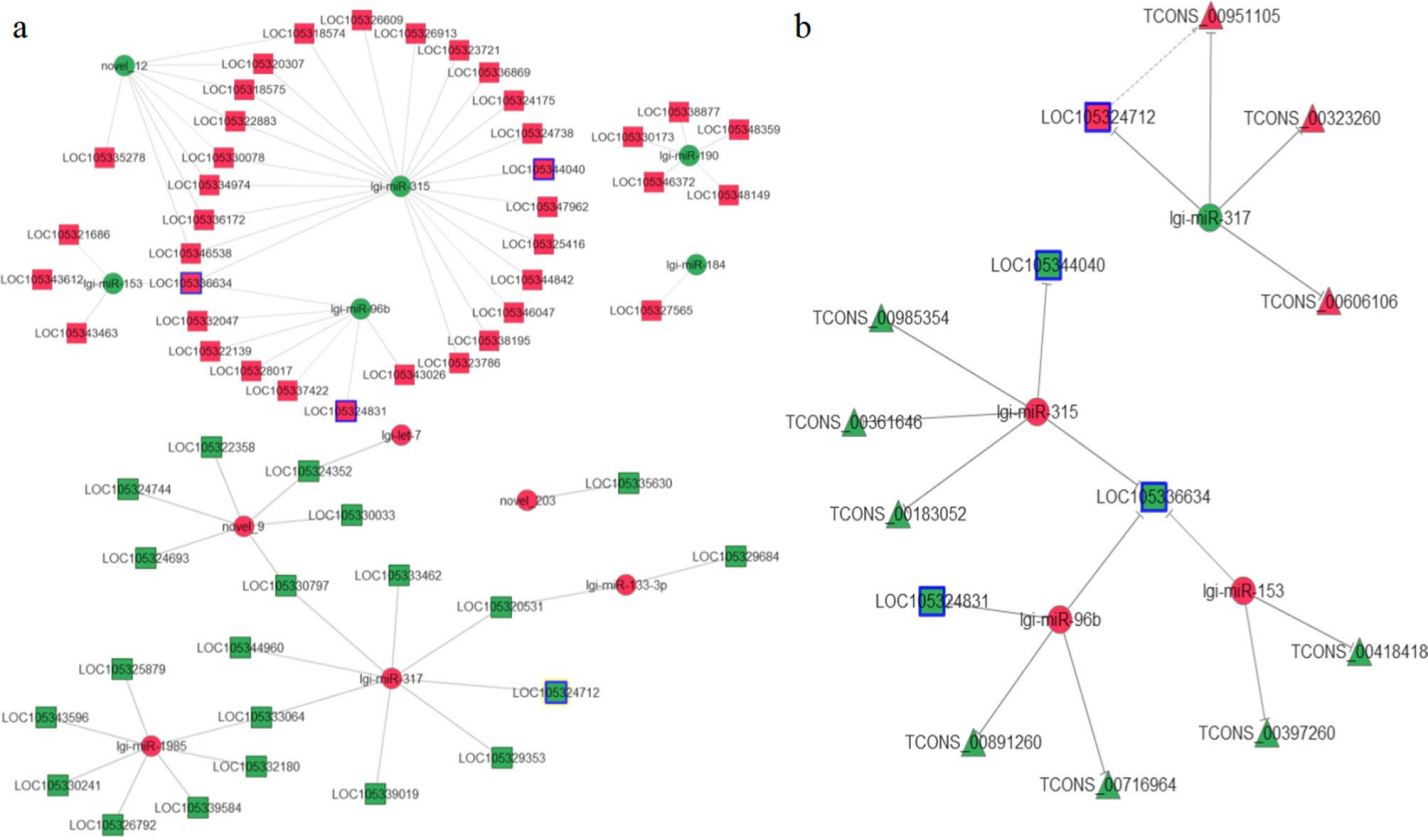
Interaction network of the differentially expressed miRNAs and their targets. (**a**) The miRNA–mRNA interaction network in WS vs NS comparison. (**b**) The lncRNA–miRNA–mRNA interaction network in NS vs WS comparison. The miRNAs are represented in circle, genes are represented in rectangle, lncRNAs are represented in triangle. The up-regulation expression is shown in red, the down-regulation expression is shown in green. The miRNA of PTM < 1000 and transcripts of RPKM < 10 was filtered.

In WS vs NS comparison, 22 up-regulated miRNAs were predicted to negatively regulate 136 down-regulated mRNAs, 8 down-regulated miRNAs were predicted to negatively regulate 80 up-regulated mRNAs (Supplementary Table S4). The miRNA-target regulatory network was represented after filtering of miRNA of TPM < 1000 (Fig. 5a). In the interacting network, lgi-miR-317 seemed to play a central role and down-regulated the expression level of *peroxidase* (LOC105324712) in WS vs NS comparison.

### The lncRNA–miRNA–mRNA networks related to pigmentation

Six previously reported pigmentation related genes in oyster were predicted to be targets by 37 miRNAs (Supplementary Table S5). For the six pigmentation related genes, the negative correlation between miRNA and its target gene was only found in the WS vs NS comparison. And four of six genes were identified to be negatively regulated by four conserved miRNA (Table 2). So four miRNAs were suggested to involve in shell pigmentation. The four miRNAs were predicted to negatively regulate ten target lncRNAs (Fig. 5b).

**Table 2:** The list of differentially expressed miRNAs and their target genes involving in pigmentation.

iRNA	WS_TPM	NM_TPM	log_2_ (Fold_change)	*p*.value	Target gene ID/description	Related pigmentation
lgi-miR-315	7539.41	21,664.64	− 1.5228	0	LOC105344040/tyrosinase-like protein 2	Melanin
					LOC105336634/cytochrome P450 2U1	Carotenoid, melanin, tetrapyrrole
lgi-miR-96b	7532.58	18,149.39	− 1.2687	0	LOC105324831/tyrosinase-like protein 3	Melanin
					LOC105336634/cytochrome P450 2U1	Carotenoid, melanin, tetrapyrrole
lgi-miR-317	4337.90	1397.99	1.6336	0	LOC105324712/peroxidase	Melanin, tetrapyrrole,
lgi-miR-153	326.03	1020.39	− 1.646	1.82E−66	LOC105336634/cytochrome P450 2U1	Carotenoid, melanin, tetrapyrrole

*WS* white shell oysters, *NS* partially pigmented shell oysters, *TPM* the tag counts per million aligned miRNAs.

### Validation and expression analysis of identified miRNAs

**Figure 6:**
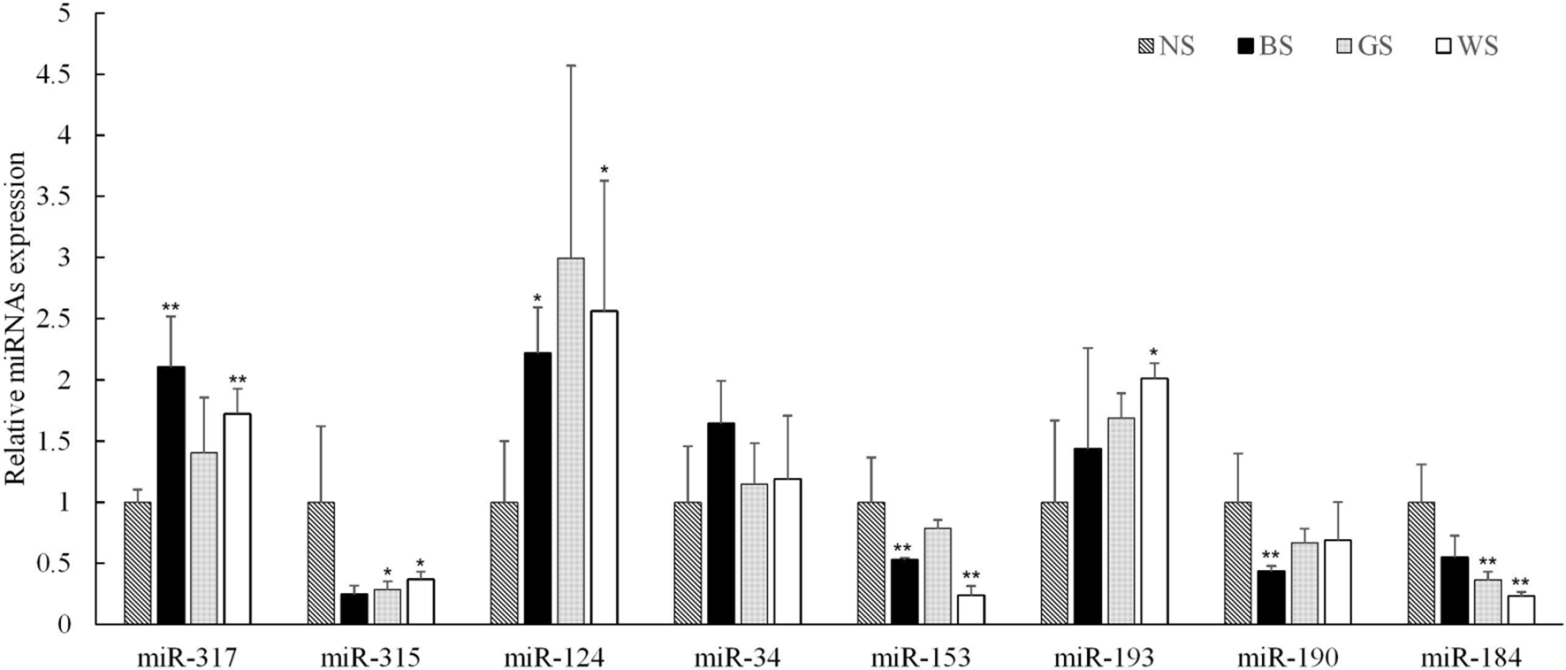
Expression of miRNAs quantified with qRT-PCR. Eight differentially expressed miRNAs were examined in the four shell colors families of *Crassostrea gigas*. Values are shown as mean ± SD (*n* = 3), *, P < 0.05, **, P < 0.01.

A total of eight DE miRNAs were selected to determine their expression levels in four samples by qRT-PCR. The results of qRT-PCR analysis showed that the expression patterns of these miRNAs were consistent with the deep sequencing results (Fig. 6). Using qRT-PCR, the expression level of lgi-miRNA-317, *peroxidase* (LOC_105324712, XM_011423866) and its potential *cis*-acting lincRNA (TCONS_00951105) was studied, lgi-miRNA-317 showed opposite expression with XM_011423866 and TCONS_00951105 in WS vs NS comparison^18^.

## Discussion

In eukaryotes, miRNAs have been reported to influence diverse biological process including animal body coloration, their role in post-transcriptional regulation were important. With the developing of the high-throughput sequencing technologies, studies on miRNA transcriptome profiles in various mollusks have been emerging^9,15,19,20,33,34^. However, little information is available on the miRNAs and miRNAs targets underlying shell color polymorphism^15^. In *C. gigas*, the shell color was a trait to improve product value, which was regulated by intricate molecular mechanism. In this study, we reported the miRNA transcriptome in mantle from four shell color variants of *C. gigas* using high-throughput technology, and identified the key miRNAs and functional miRNA-targets in shell color formation using integrated analysis of miRNA and mRNA expression profiles. This study provided a foundation for future functional studies on the relationship between miRNA expression and development and function of shell including shell color.

Reliable and abundant sRNAs database in mantle of *C. gigas* was produced for downstream miRNA analysis by high-throughput sequencing. The majority of the sRNAs were 21–23 nt in length (Fig. 1), which is characteristic of sRNAs from metazoans^11,35^. The most frequent length of sRNA sequenced was 22 nt, which is identical to the classical size of Dicer cleavage products^35^, suggesting the conservation of miRNAs. The proportion of total rRNA was only 1.7%, which was much less than 40%, indicating high quality data obtained for downstream analysis in this work^36^.

A total of 53 conserved miRNAs in all four mantle libraries were grouped into 38 miRNA families according to the new miRNA family classifications in Rfam. Most of these identified miRNA families in *C. gigas* are also conserved in other molluscan species, such as *Pinctada martensii*^37^, *Littorina littorea*^38^, *Haliotis discus hannai*^34^ and *Pinctada fucata*^33^. And let-7, mir-9, mir-315, mir-10, mir-137, mir-29, mir-133, mir-2 families can be found in most molluscan species, suggesting they are very conserved. A total 91 novel miRNAs were identified, most of them represented rare expression, which suggested they are hard to be identified. Most of miRNA (114/144) were stably identified in each library, indicating the sequenced miRNAs were reliable.

The DE miRNAs are identified, which are promising to regulate the shell color polymorphism. DE miRNAs represent differential expression pattern in NS sample (Fig. 2), which is consistent with the pronounced phenotypic difference between NS and any of other three. Shells in NS family show pigmentation strip, while the shells in other three families represent solid pigmentation distribution^16^. The pigmentation strip is generally black or a mixture of black and other colors. While the solid pigmentation represented one color in a shell, which could be black, white or yellow. A total of 15 DE miRNAs are shared by WS vs NS, GS vs NS and BS vs NS comparisons, including ten conserved miRNA of lgi-miR-96b, lgi-miR-745b, lgi-miR-34, lgi-miR-317, lgi-miR-315, lgi-miR-2c, lgi-miR-281-3p, lgi-miR-193, lgi-miR-153 and lgi-miR-124. In mouse melanocytes, miR-193 has been detected to downregulate after stimulating the product, processing, and transport of melanosomes^39^. Both miR-34 and miR-124 have been detected to influence migration of retinal pigment epithelium^40,41^. And the result suggested that more DE miRNAs are involving the represent of shell pigmentation strip than the represent of shell solid pigmentation. The functional role of such DE miRNAs is the focus of future investigation.

In animals, miRNAs target mRNAs with partial sequence complementarity, which could be used to analysis putative specific genes targeted by these miRNAs. Based on KEGG pathway analysis, theses targeted genes mainly involved in the ECM-receptor interaction and Notch signaling pathway, which have been reported in mantle transcriptome analysis. Notch signaling pathway was identified in WS vs BS, which had been reported to regulate shell pigmentation in both *C. gigas*^17^ and *Meretrix meretrix*^42^. The function enrichment analysis further confirmed the miRNAs played a role in shell pigmentation.

The major physiological difference of interest between the samples analyzed was shell color, so we focused on potential target genes regulating pigmentation. A total of six genes were reported to involve in shell pigmentation^18^, of which four genes were predicted to be negatively regulated by four miRNAs. Then the lncRNA–miRNA–mRNA networks were constructed in NS vs WS comparison by integrating previously reported lncRNA transcriptome (Fig. 5). And it is noteworthy, both the *peroxidase* (LOC_105324712, XM_011423866) and its potential *cis*-acting lincRNA TCONS_00951105 were negatively regulated by lgi-miR-317. The *peroxidase* (LOC_105324712) has been reported to regulate the black pigmentation in oyster shell^43^. And lgi-miR-317 was reported to express at the body pigmentation stage in silkworm larvae^44^. So the TCONS_00951105-lgi-miR-317-XM_011423866 might act as the ceRNA networks and play a key role in black shell pigmentation, which remain to be confirmed by more experiment. The *Cytochrome P450 2U1* was identified to be negatively regulated by lgi-miR-315, lgi-miR-96b and lgi-miR-153, which suggested a fine regulation in quantity involving carotenoid, melanin, and tetrapyrrole in NS group. The miR-153 was reported to act as a ceRNA with the lncRNA KCNQ1OTQ to depress the expression of *receptor tyrosine kinase* to inhibit melanogenesis^45^. So, lgi-miR-153 are promising to act in ceRNA network for regulating melanin in oyster. The *Tyrosinase-like protein 3* (LOC105324831) and *Tyrosinase-like protein 2* (LOC105344040), reported as the key enzyme to regulate melanin synthesis, were negatively regulated by lgi-miR-96b and lgi-miR-315, respectively. Altogether, our studies strongly suggested that DE miRNAs and their targets play an important role in pigmentation. And lgi-miR-317 might act as a ceRNA to de-repress the expression of *peroxidase* (LOC_105324712, XM_011423866) and its potential *cis*-acting lincRNA TCONS_00951105 to regulate melanin synthesis, by binding to these two transcript.

## Conclusion

The miRNAs have emerged as important roles in the regulation of gene expression. These DE-miRNAs and their targets might further be used as molecular markers to screen for shell color variant of oyster. In summary, this research is the first analysis of miRNAs profiles involving shell pigmentation in *C. gigas* using the Illumina HiSeq sequencing platform. A total of 144 miRNAs were identified, 47 miRNAs were detected to differentially express. And four DE miRNAs (lgi-miR-315, lgi-miR-96b, lgi-miR-317 and lgi-miR-153) were closely associated with shell pigmentation. In addition, we identified the lgi-miR-317 and its targets mRNA *peroxidase* and lncRNA TCONS_00951105 might act as the ceRNA networks and play a key role in shell melanin synthesis and conformed their dynamic expression pattern in four oyster shell color variants. Taken together, our findings provide useful information for understanding the molecular mechanism of shell pigmentation and enrich the knowledge of miRNAs in marine invertebrate. These findings will further be used to improve artificial selection efficiency and contribute to the genetic improvements of the oyster aquaculture.

## Supplementary information

Supplementary Tables.
