# Supplementary material for: Integrated analysis of microRNA and mRNA expression profiles in Crassostrea gigas to reveal functional miRNA and miRNA-targets regulating shell pigmentation
Source: Sci Rep. 2020 Nov 19;10:20238. doi: 10.1038/s41598-020-77181-0 (PMC7678851; doi:10.1038/s41598-020-77181-0)
Supplement: Supplementary file 1 — Supplementary Tables. [file 41598_2020_77181_MOESM1_ESM.pdf]

Supplementary Information (SI)

Integrated analysis of microRNA and mRNA expression profiles in *Crassostrea gigas* to reveal functional miRNA and miRNA-targets regulating shell pigmentation

Dandan Feng[1] · Qi Li[1,2*] · Hong Yu[1] · Shikai Liu[1] · Lingfeng Kong[1] · Shaojun Du[3]

1 Key Laboratory of Mariculture, Ministry of Education, Ocean University of China, Qingdao 266003, China

2 Laboratory for Marine Fisheries Science and Food Production Processes, Qingdao National Laboratory for Marine Science and Technology, Qingdao 266237, China

3 Institute of Marine and Environmental Technology, Department of Biochemistry and Molecular Biology, University of Maryland School of Medicine, Baltimore, MD, United States

*corresponding author: qili66@ouc.edu.cn

Supplementary Table S1 Primers used in qRT-PCR

Small RNA	Stem-Loop Primer	qRT-PCR Forward Primer
lgi-miR-317	CTCAACTGGTGTCGTGGAGTCGGCAATTCAGTTGAGAGAAGATA	ACACTCCAGCTGGGTGAACACAGCTGGTGG
lgi-miR-315	CTCAACTGGTGTCGTGGAGTCGGCAATTCAGTTGAGGGCTTTCT	ACACTCCAGCTGGGTTTTGATTGTTGCTC
lgi-miR-124	CTCAACTGGTGTCGTGGAGTCGGCAATTCAGTTGAGTGGCATT	ACACTCCAGCTGGGTAAGGCACGCGGTG
lgi-miR-34	CTCAACTGGTGTCGTGGAGTCGGCAATTCAGTTGAGACTACCAG	ACACTCCAGCTGGGTGGCAGTGTGGTTAG
lgi-miR-153	CTCAACTGGTGTCGTGGAGTCGGCAATTCAGTTGAGGATCACTT	ACACTCCAGCTGGGTTGCATAGTCACAA
lgi-miR-193	CTCAACTGGTGTCGTGGAGTCGGCAATTCAGTTGAGGTTGGGAT	ACACTCCAGCTGGGTACTGGCCTGCAAA
lgi-miR-190	CTCAACTGGTGTCGTGGAGTCGGCAATTCAGTTGAGACCAATTA	ACACTCCAGCTGGGAGATATGTTTGATA
lgi-miR-184	CTCAACTGGTGTCGTGGAGTCGGCAATTCAGTTGAGGCCCTTAT	ACACTCCAGCTGGGTGGACGGAGAACTG
miRNA reverse primer	TGGTGTCGTGGAGTCG	
U6 forward primer	TGGAACGATACAGAGAAGATTAGC	
U6 reverse primer	TATGGAACGCTTCACGAATTT	

Supplementary Table 2 The abundance and family of miRNAs expressed in the four libraries

sRNA	BSM.readcount	WSM.readcount	GSM.readcount	NSM.readcount	BSM.tpm	WSM.tpm	GSM.tpm	NSM.tpm	SUM tpm	Family
lgi-miR-100	1004454	807645	1218791	1576800	254295.69	226040.32	266534.65	287117.41	480336.01	mir-10
lgi-miR-1	524594	486046	590814	737992	132810.46	136032.53	129203.78	134379.98	268842.99	mir-1
lgi-miR-10	324625	390913	418068	729652	82184.69	109407.10	91426.35	132861.36	191591.79	mir-10
lgi-miR-184	269459	185600	235092	517018	68218.42	51944.96	51411.74	94143.12	120163.37	mir-184
lgi-miR-279	286539	199320	325829	137407	72542.53	55784.85	71254.81	25020.26	128327.38	mir-279
novel_3	205903	179345	260515	122063	52128.07	50194.33	56971.44	22226.29	102322.40	
novel_1	144111	140213	164118	191949	36484.31	39242.23	35890.59	34951.74	75726.54	
lgi-miR-8	136353	112729	160376	191092	34520.23	31550.12	35072.27	34795.69	66070.35	mir-8
lgi-miR-7	102959	112811	101438	214798	26065.93	31573.07	22183.25	39112.28	57639.01	mir-7
novel_203	130458	124352	93511	61855	33027.80	34803.12	20449.71	11263.09	67830.92	
lgi-miR-9-5p	87414	80876	100637	136537	22130.43	22635.24	22008.08	24861.84	44765.67	mir-9
lgi-miR-133-3p	88199	107312	127841	52921	22329.17	30034.04	27957.26	9636.31	52363.21	mir-133
lgi-let-7	103631	101442	93394	67600	26236.06	28391.16	20424.12	12309.19	54627.23	let-7
novel_9	68298	90939	75810	49459	17290.87	25451.63	16578.72	9005.92	42742.50	
novel_210	40757	33117	51230	80884	10318.37	9268.65	11203.37	14728.06	19587.02	
lgi-miR-315	33526	28721	38642	111594	8487.71	8038.31	8450.53	20320.00	16526.03	mir-315
lgi-miR-96b	32295	28695	43212	93487	8176.06	8031.04	9449.93	17022.92	16207.10	
lgi-miR-1994a	29792	32743	51495	29904	7542.38	9163.97	11261.33	5445.18	16706.36	mir-1994
lgi-miR-1985	30680	39434	34971	26576	7767.20	11036.62	7647.73	4839.19	18803.82	
lgi-miR-67	28133	22996	42288	26272	7122.38	6436.02	9247.87	4783.83	13558.40	mir-67
novel_12	19247	15349	21610	70062	4872.73	4295.81	4725.84	12757.50	9168.54	
lgi-miR-216b	23660	24139	41923	25940	5989.96	6755.92	9168.05	4723.38	12745.88	mir-216
lgi-miR-29	20239	18018	21964	22287	5123.87	5042.80	4803.26	4058.21	10166.67	mir-29
lgi-miR-31	15705	18166	21549	17811	3976.00	5084.22	4712.50	3243.18	9060.23	mir-31
novel_18	19648	16609	21371	14727	4974.25	4648.46	4673.58	2681.62	9622.70	
lgi-miR-96a	15898	12866	20680	26281	4024.87	3600.88	4522.46	4785.47	7625.75	
lgi-miR-1994b	14394	15866	22100	15434	3644.10	4440.51	4833.00	2810.36	8084.61	mir-1994
lgi-miR-317	16537	16525	19974	7201	4186.64	4624.95	4368.07	1311.22	8811.59	
novel_23	13598	10982	14092	14962	3442.58	3073.60	3081.75	2724.41	6516.18	
novel_27	16935	7449	11876	17595	4287.40	2084.80	2597.14	3203.85	6372.20	
lgi-miR-745b	11780	16857	15011	4257	2982.32	4717.87	3282.72	775.15	7700.19	
novel_25	11679	12135	13356	13352	2956.75	3396.29	2920.79	2431.25	6353.04	

lgi-miR-2d	10550	9411	12432	7191	2670.92	2633.91	2718.73	1309.40	5304.83	
novel_30	7710	11207	11015	4167	1951.93	3136.57	2408.85	758.76	5088.49	
novel_33	7733	6710	8062	5496	1957.75	1877.97	1763.06	1000.76	3835.72	
lgi-miR-216a	5391	3844	5756	10131	1364.83	1075.84	1258.77	1844.74	2440.67	mir-216
novel_41	4129	4857	5929	4449	1045.33	1359.36	1296.60	810.11	2404.69	
novel_22	4201	4521	6966	3580	1063.56	1265.32	1523.38	651.88	2328.88	
novel_38	2174	3864	6388	7613	550.39	1081.44	1396.98	1386.24	1631.83	
lgi-miR-2001	2895	3032	4948	9120	732.92	848.58	1082.07	1660.65	1581.51	mir-2001
lgi-miR-281-3p	5197	4852	5723	1750	1315.71	1357.96	1251.55	318.66	2673.67	mir-46
lgi-miR-2c	2478	3188	3185	1108	627.35	892.24	696.52	201.75	1519.59	mir-2
lgi-miR-153	2009	1242	2341	5256	508.61	347.61	511.95	957.06	856.22	mir-153
novel_205	1958	2703	2861	2328	495.70	756.50	625.67	423.90	1252.21	
lgi-miR-193	2064	2265	3318	691	522.54	633.92	725.61	125.82	1156.46	
lgi-miR-745a	1680	2823	2460	1062	425.32	790.09	537.97	193.38	1215.41	
lgi-miR-1175-3p	2407	1098	2066	2717	609.38	307.30	451.81	494.73	916.68	mir-1175
novel_8	1163	1081	1330	3188	294.43	302.55	290.85	580.50	596.98	
lgi-miR-12	1463	1289	1810	1008	370.38	360.76	395.82	183.55	731.14	
novel_52	1311	1444	1358	1505	331.90	404.14	296.98	274.04	736.04	
novel_48	1126	1146	1273	2300	285.07	320.74	278.39	418.80	605.80	
novel_55	898	1477	924	2117	227.34	413.38	202.07	385.48	640.72	
novel_60	1429	1282	849	1252	361.78	358.80	185.67	227.98	720.58	
lgi-miR-1986	1005	1305	917	574	254.43	365.24	200.54	104.52	619.67	mir-1986
novel_66	986	692	733	1058	249.62	193.67	160.30	192.65	443.30	
lgi-miR-34	772	1085	701	313	195.45	303.67	153.30	56.99	499.11	mir-34
novel_24	636	631	718	839	161.01	176.60	157.02	152.77	337.62	
lgi-miR-1990	665	565	676	921	168.36	158.13	147.83	167.70	326.49	mir-1990
novel_69	657	576	656	676	166.33	161.21	143.46	123.09	327.54	
lgi-miR-9-3p	455	439	504	704	115.19	122.87	110.22	128.19	238.06	mir-9
lgi-miR-278	375	494	509	655	94.94	138.26	111.31	119.27	233.20	
novel_71	471	1011	13	158	119.24	282.95	2.84	28.77	402.20	
lgi-miR-1992	192	243	485	492	48.61	68.01	106.06	89.59	116.62	mir-1992
lgi-miR-124	322	429	411	80	81.52	120.07	89.88	14.57	201.59	mir-124
lgi-miR-87	262	314	334	178	66.33	87.88	73.04	32.41	154.21	mir-87
novel_76	405	182	346	109	102.53	50.94	75.67	19.85	153.47	
novel_73	158	301	199	210	40.00	84.24	43.52	38.24	124.24	

novel_83	259	325	5	37	65.57	90.96	1.09	6.74	156.53	
lgi-miR-183	118	125	70	159	29.87	34.98	15.31	28.95	64.86	mir-263
novel_84	161	63	57	176	40.76	17.63	12.47	32.05	58.39	
lgi-miR-71	51	46	73	79	12.91	12.87	15.96	14.39	25.79	mir-71
novel_190	2	85	60	95	0.51	23.79	13.12	17.30	24.30	
lgi-miR-981	61	72	40	13	15.44	20.15	8.75	2.37	35.59	mir-981
lgi-miR-33-5p	33	48	72	27	8.35	13.43	15.75	4.92	21.79	mir-33
novel_96	45	33	50	32	11.39	9.24	10.93	5.83	20.63	
novel_263	31	49	29	14	7.85	13.71	6.34	2.55	21.56	
lgi-miR-1175-5p	34	16	45	16	8.61	4.48	9.84	2.91	13.09	mir-1175
novel_79	29	33	15	24	7.34	9.24	3.28	4.37	16.58	
lgi-miR-190	15	6	16	80	3.80	1.68	3.50	14.57	5.48	mir-190
novel_207	21	57	0	0	5.32	15.95	0.00	0.00	21.27	
lgi-miR-92	20	23	27	19	5.06	6.44	5.90	3.46	11.50	mir-25
novel_106	9	17	24	14	2.28	4.76	5.25	2.55	7.04	
novel_271	11	18	19	15	2.78	5.04	4.16	2.73	7.82	
novel_188	29	9	17	1	7.34	2.52	3.72	0.18	9.86	
novel_267	1	3	54	2	0.25	0.84	11.81	0.36	1.09	
novel_108	13	13	10	22	3.29	3.64	2.19	4.01	6.93	
novel_186	5	15	9	26	1.27	4.20	1.97	4.73	5.46	
novel_270	13	8	21	9	3.29	2.24	4.59	1.64	5.53	
novel_104	1	0	46	0	0.25	0.00	10.06	0.00	0.25	
lgi-miR-242a	4	27	1	6	1.01	7.56	0.22	1.09	8.57	mir-242_2
lgi-miR-137	5	13	14	9	1.27	3.64	3.06	1.64	4.90	mir-137
novel_242	3	8	15	16	0.76	2.24	3.28	2.91	3.00	
lgi-miR-750	6	10	11	5	1.52	2.80	2.41	0.91	4.32	
lgi-miR-182	9	4	7	14	2.28	1.12	1.53	2.55	3.40	mir-182
novel_56	12	3	14	1	3.04	0.84	3.06	0.18	3.88	
novel_111	7	6	7	11	1.77	1.68	1.53	2.00	3.45	
novel_109	4	5	3	16	1.01	1.40	0.66	2.91	2.41	
lgi-miR-252b	5	5	10	5	1.27	1.40	2.19	0.91	2.67	mir-252
novel_129	2	0	23	0	0.51	0.00	5.03	0.00	0.51	
novel_128	3	4	9	9	0.76	1.12	1.97	1.64	1.88	
novel_131	3	10	5	2	0.76	2.80	1.09	0.36	3.56	
novel_209	5	2	14	0	1.27	0.56	3.06	0.00	1.83	

novel_112	15	0	2	0	3.80	0.00	0.44	0.00	3.80
novel_138	6	3	2	6	1.52	0.84	0.44	1.09	2.36
novel_120	7	1	2	7	1.77	0.28	0.44	1.27	2.05
novel_136	6	4	3	2	1.52	1.12	0.66	0.36	2.64
novel_100	7	2	0	7	1.77	0.56	0.00	1.27	2.33
novel_142	2	2	8	0	0.51	0.56	1.75	0.00	1.07
novel_54	2	2	5	3	0.51	0.56	1.09	0.55	1.07
novel_134	0	0	0	13	0.00	0.00	0.00	2.37	0.00
novel_185	5	0	0	6	1.27	0.00	0.00	1.09	1.27
novel_272	1	0	4	6	0.25	0.00	0.87	1.09	0.25
novel_199	1	3	0	6	0.25	0.84	0.00	1.09	1.09
novel_235	7	0	0	2	1.77	0.00	0.00	0.36	1.77
novel_140	0	3	4	2	0.00	0.84	0.87	0.36	0.84
novel_20	1	2	4	2	0.25	0.56	0.87	0.36	0.81
novel_211	2	0	1	6	0.51	0.00	0.22	1.09	0.51
novel_220	4	0	0	4	1.01	0.00	0.00	0.73	1.01
novel_139	0	0	0	9	0.00	0.00	0.00	1.64	0.00
novel_166	4	0	2	1	1.01	0.00	0.44	0.18	1.01
novel_162	1	1	0	6	0.25	0.28	0.00	1.09	0.53
novel_194	0	1	1	6	0.00	0.28	0.22	1.09	0.28
novel_154	4	0	0	3	1.01	0.00	0.00	0.55	1.01
novel_234	4	1	0	1	1.01	0.28	0.00	0.18	1.29
novel_135	3	1	1	1	0.76	0.28	0.22	0.18	1.04
novel_189	0	0	6	0	0.00	0.00	1.31	0.00	0.00
novel_143	1	2	0	2	0.25	0.56	0.00	0.36	0.81
novel_161	1	3	0	0	0.25	0.84	0.00	0.00	1.09
novel_259	0	0	4	1	0.00	0.00	0.87	0.18	0.00
novel_225	1	2	1	0	0.25	0.56	0.22	0.00	0.81
novel_144	0	1	3	0	0.00	0.28	0.66	0.00	0.28
novel_164	0	0	1	3	0.00	0.00	0.22	0.55	0.00
novel_228	1	1	1	0	0.25	0.28	0.22	0.00	0.53
novel_195	0	0	3	0	0.00	0.00	0.66	0.00	0.00
novel_151	0	2	0	0	0.00	0.56	0.00	0.00	0.56
novel_157	0	0	0	3	0.00	0.00	0.00	0.55	0.00
novel_173	0	1	1	0	0.00	0.28	0.22	0.00	0.28

novel_177	0	0	2	0	0.00	0.00	0.44	0.00	0.00	
novel_227	0	0	2	0	0.00	0.00	0.44	0.00	0.00	
novel_237	1	0	0	1	0.25	0.00	0.00	0.18	0.25	
novel_183	0	0	1	1	0.00	0.00	0.22	0.18	0.00	
novel_248	0	1	0	0	0.00	0.28	0.00	0.00	0.28	
novel_212	1	0	0	0	0.25	0.00	0.00	0.00	0.25	
lgi-miR-1993	0	0	0	1	0.00	0.00	0.00	0.18	0.00	mir-1993

Supplementary Table 3 The predicted targets of differentially expressed miRNAs in six pairwise comparisons

WS vs NS	BS vs NS	GS vs NS	WS vs BS	WS vs GS	BS vs GS
LOC105320548	LOC105322766	LOC105342862	LOC105322800	LOC105326589	LOC105327534
LOC105328017	LOC105331602	LOC105323605	LOC105322457	LOC105340721	LOC105323913
LOC105333636	LOC105318129	LOC105338857	LOC105338920	LOC105331123	LOC105333596
LOC105330033	LOC105321678	LOC105346732	LOC105337639	LOC105335690	LOC105325987
LOC105320181	LOC105318591	LOC105317679	LOC105341096	LOC105329095	LOC105335846
LOC105321793	LOC105321887	LOC105341974	LOC105327818	LOC105325709	LOC105319237
LOC105347962	LOC105348149	LOC105319706	LOC105335502	LOC105347683	LOC105326362
LOC105327417	LOC105328042	LOC105318805	LOC105345077	LOC105317941	LOC105341116
LOC105336711	LOC105317776	LOC105335027	LOC105337008	LOC105326853	LOC105336823
LOC105330079	LOC105337616	LOC105334645	LOC105337755	LOC105346062	LOC105334794
LOC105323721	LOC105323605	LOC105328889	LOC105335370	LOC105334215	LOC105329622
LOC105338828	LOC105333401	LOC105337763	LOC105341286	LOC105334815	LOC105341947
LOC105345500	LOC105343463	LOC105333401	LOC105341947	LOC105326786	LOC105321691
LOC105322358	LOC105328052	LOC105346405	LOC105324599	LOC105325995	LOC105322294
LOC105341599	LOC105337541	LOC105342031	LOC105347740	LOC105322691	LOC105317819
LOC105344958	LOC105348436	LOC105328052	LOC105318724	LOC105333401	LOC105325189
LOC105324874	LOC105321986	LOC105344999	LOC105345824	LOC105327479	LOC105327399
LOC105318835	LOC105328889	LOC105340025	LOC105320704	LOC105322213	LOC105330498
LOC105322139	LOC105342031	LOC105340526	LOC105343612	LOC105322210	LOC105320131
LOC105324744	LOC105346405	LOC105343463	LOC105319327	LOC105335403	LOC105346832
LOC105324693	LOC105334645	LOC105340464	LOC105340251	LOC105322025	LOC105321555
LOC105318243	LOC105318430	LOC105337521	LOC105319299	LOC105340628	LOC105327681
LOC105323666	LOC105340553	LOC105336617	LOC105326779	LOC105321788	LOC105318591
LOC105324352	LOC105328522	LOC105325108	LOC105341807	LOC105327681	LOC105346199
LOC105330797	LOC105346480	LOC105317242	LOC105333700	LOC105337509	LOC105330789
LOC105346446	LOC105328869	LOC105321257	LOC105319473	LOC105325625	LOC105345395
LOC105321494	LOC105345788	LOC105333271	LOC105319237	LOC105343792	LOC105335005
LOC105335061	LOC105346732	LOC105318801	LOC105320319	LOC105340464	LOC105332469
LOC105325790	LOC105349162	LOC105329196	LOC105328176	LOC105344999	LOC105347749
LOC105322065	LOC105346734	LOC105338945	LOC105346734	LOC105343641	LOC105338306
LOC105328789	LOC105327947	LOC105331123	LOC105329557	LOC105321958	LOC105325108
LOC105331147	LOC105322148	LOC105327836	LOC105323913	LOC105337718	LOC105326779

LOC105319299	LOC105334082	LOC105326899	LOC105333596	LOC105317220	LOC105319693
LOC105328379	LOC105318316	LOC105342691	LOC105333997	LOC105326650	LOC105330656
LOC105320531	LOC105321257	LOC105322025	LOC105338351	LOC105317555	LOC105349002
LOC105329570	LOC105327547	LOC105327036	LOC105318847	LOC105338828	LOC105321536
LOC105346301	LOC105339864	LOC105332297	LOC105336942	LOC105336368	LOC105318243
LOC105319189	LOC105329133	LOC105330016	LOC105330818	LOC105323237	LOC105322065
LOC105340984	LOC105338243	LOC105345325	LOC105349083	LOC105321454	LOC105341852
LOC105339019	LOC105344085	LOC105328789	LOC105337069	LOC105330446	LOC105328916
LOC105333064	LOC105347327	LOC105326609	LOC105335413	LOC105327471	LOC105338770
LOC105335743	LOC105338410	LOC105337016	LOC105341159	LOC105338466	LOC105343695
LOC105348016	LOC105341643	LOC105337422	LOC105348023	LOC105330656	LOC105322231
LOC105346372	LOC105340098	LOC105323254	LOC105334074	LOC105322065	LOC105344085
LOC105343049	LOC105340237	LOC105330790	LOC105343792	LOC105337521	LOC105317600
LOC105336820	LOC105328612	LOC105335630	LOC105340894	LOC105326362	LOC105326899
LOC105318316	LOC105338770	LOC105329996	LOC105319578	LOC105343996	LOC105348493
LOC105327613	LOC105332779	LOC105337509	LOC105331418	LOC105342859	LOC105320490
LOC105328052	LOC105326059	LOC105344724	LOC105320241	LOC105319151	LOC105340237
LOC105328612	LOC105330100	LOC105332469	LOC105336134	LOC105319578	LOC105329676
LOC105321610	LOC105323026	LOC105348507	LOC105327547	LOC105322021	LOC105344999
LOC105333462	LOC105344105	LOC105334049	LOC105346832	LOC105345273	LOC105348617
LOC105329353	LOC105342437	LOC105338466	LOC105331837	LOC105339873	LOC105318367
LOC105343654	LOC105327471	LOC105323986	LOC105327204	LOC105346575	LOC105347561
LOC105326239	LOC105340774	LOC105319642	LOC105332254	LOC105332921	LOC105335829
LOC105335667	LOC105335722	LOC105321536	LOC105340721	LOC105338505	LOC105329193
LOC105330238	LOC105342484	LOC105332542	LOC105336661	LOC105335729	LOC105326615
LOC105348744	LOC105323803	LOC105330656	LOC105343024	LOC105332653	LOC105317288
LOC105330790	LOC105327818	LOC105328379	LOC105327036	LOC105346061	LOC105348632
LOC105347258	LOC105326030	LOC105327471	LOC105332808	LOC105319193	LOC105327837
LOC105323254	LOC105332808	LOC105320778	LOC105330079	LOC105327837	LOC105329196
LOC105335277	LOC105330954	LOC105319151	LOC105344883	LOC105334651	LOC105335743
LOC105331837	LOC105324803	LOC105319578	LOC105319479	LOC105334131	LOC105331005
LOC105344960	LOC105330313	LOC105344225	LOC105322743	LOC105324414	LOC105343767
LOC105321418	LOC105339666	LOC105326362	LOC105319184	LOC105323721	LOC105333649
LOC105324712	LOC105334246	LOC105325902	LOC105318243	LOC105343393	LOC105345359
LOC105337211	LOC105317412	LOC105333692	LOC105319458	LOC105337238	LOC105332297

LOC105326914	LOC105324719	LOC105347171	LOC105324598	LOC105328916	LOC105336665
LOC105342435	LOC105333946	LOC105320494	LOC105336627	LOC105331602	LOC105348133
LOC105331979	LOC105344924	LOC105346882	LOC105318519	LOC105347740	LOC105340890
LOC105317540	LOC105344244	LOC105326757	LOC105328415	LOC105345812	LOC105333532
LOC105328030	LOC105318571	LOC105338421	LOC105324307	LOC105348493	LOC105343026
LOC105331186	LOC105335448	LOC105338930	LOC105327399	LOC105328358	LOC105337688
LOC105342697	LOC105317472	LOC105325257	LOC105330109	LOC105335277	LOC105337509
LOC105344419	LOC105324796	LOC105333064	LOC105337186	LOC105341807	LOC105331882
LOC105347939	LOC105322705	LOC105318605	LOC105320805	LOC105340527	LOC105344552
LOC105349096	LOC105333814	LOC105324897	LOC105338243	LOC105341715	LOC105324437
LOC105343596	LOC105328434	LOC105333239	LOC105317690	LOC105332332	LOC105339219
LOC105326913	LOC105348287	LOC105319071	LOC105324897	LOC105335743	LOC105348594
LOC105341999	LOC105318799	LOC105335829	LOC105330416	LOC105330173	LOC105343500
LOC105338930	LOC105338725	LOC105326795	LOC105342435	LOC105330034	LOC105335061
LOC105343857	LOC105346199	LOC105333421	LOC105334682	LOC105338263	LOC105322771
LOC105340900	LOC105318461	LOC105328891	LOC105324953	LOC105340240	LOC105336627
LOC105327657	LOC105327817	LOC105344149	LOC105337611	LOC105348052	LOC105345054
LOC105320494	LOC105326391	LOC105345520	LOC105328316	LOC105337611	LOC105329979
LOC105338945	LOC105331146	LOC105346062	LOC105338615	LOC105346685	LOC105321872
LOC105328916	LOC105325987	LOC105343641	LOC105318241	LOC105326413	LOC105339960
LOC105335624	LOC105319380	LOC105336978	LOC105325250	LOC105325189	LOC105334682
LOC105338112	LOC105325178	LOC105348133	LOC105318972	LOC105322992	LOC105328415
LOC105340527	LOC105326918	LOC105319193	LOC105326267	LOC105344845	LOC105348656
LOC105319380	LOC105321555	LOC105327481	LOC105339960	LOC105346534	LOC105331123
LOC105343676	LOC105334682	LOC105325416	LOC105339219	LOC105338229	LOC105324307
LOC105335630	LOC105333227	LOC105330078	LOC105322753	LOC105330894	LOC105342750
LOC105328971	LOC105342078	LOC105344031	LOC105322771	LOC105334068	LOC105324632
LOC105332843	LOC105321902	LOC105322800	LOC105322857	LOC105348110	LOC105325465
LOC105318571	LOC105321886	LOC105323384	LOC105334125	LOC105348387	LOC105343234
LOC105334348	LOC105322457	LOC105330864	LOC105343500	LOC105326707	LOC105341948
LOC105339221	LOC105347610	LOC105332888	LOC105329979	LOC105342688	LOC105318972
LOC105327510	LOC105320676	LOC105326056	LOC105325299	LOC105344122	LOC105318555
LOC105332880	LOC105329159	LOC105322457	LOC105319573	LOC105320664	LOC105340209
LOC105335309	LOC105326615	LOC105347510	LOC105327471	LOC105344470	LOC105346534
LOC105332743	LOC105327534	LOC105325976	LOC105331602	LOC105318805	LOC105322640

LOC105346652	LOC105343767	LOC105332364	LOC105341948	LOC105346764	LOC105340628
LOC105326473	LOC105339751	LOC105332831	LOC105321872	LOC105343433	LOC105326059
LOC105340509	LOC105330790	LOC105323677	LOC105335690	LOC105340974	LOC105320764
LOC105335502	LOC105332934	LOC105339383	LOC105326393	LOC105320548	LOC105322025
LOC105336634	LOC105332047	LOC105323803	LOC105335061	LOC105325441	LOC105321890
LOC105339584	LOC105319670	LOC105326615	LOC105325441	LOC105339019	LOC105326056
LOC105333283	LOC105323057	LOC105345733	LOC105346534	LOC105337688	LOC105326387
LOC105337639	LOC105348370	LOC105334200	LOC105331186	LOC105319300	LOC105340464
LOC105332180	LOC105339609	LOC105347962	LOC105325710	LOC105332408	LOC105342368
LOC105325879	LOC105345054	LOC105322048	LOC105343026	LOC105317622	LOC105320548
LOC105319286	LOC105320778	LOC105335288	LOC105328522	LOC105317472	LOC105345719
LOC105330078	LOC105339775	LOC105348350	LOC105328759	LOC105332843	LOC105348149
LOC105327479	LOC105317071	LOC105321887	LOC105328914	LOC105342031	LOC105326792
LOC105332036	LOC105329230	LOC105334348	LOC105332843	LOC105329492	LOC105342688
LOC105328891	LOC105318575	LOC105330540	LOC105331882	LOC105328088	LOC105332808
LOC105317200	LOC105330389	LOC105332880	LOC105319300	LOC105321887	LOC105344845
LOC105329879	LOC105340600	LOC105320079	LOC105333532	LOC105333372	LOC105333647
LOC105318336	LOC105318574	LOC105339837	LOC105340890	LOC105334630	LOC105339019
LOC105340774	LOC105348422	LOC105335843	LOC105328088	LOC105319458	LOC105331042
LOC105327372	LOC105323529	LOC105318571	LOC105346061	LOC105335213	LOC105319923
LOC105330241	LOC105317414	LOC105317776	LOC105317328	LOC105335370	LOC105322992
LOC105339129	LOC105324272	LOC105324842	LOC105321887	LOC105341374	LOC105346764
LOC105326792	LOC105348632	LOC105330389	LOC105337833	LOC105321689	
LOC105337364	LOC105349024	LOC105348647	LOC105337688	LOC105330078	
LOC105318575	LOC105323578	LOC105328901		LOC105348133	
LOC105335690	LOC105330078	LOC105342355			
LOC105323803	LOC105333596	LOC105322303			
LOC105339136	LOC105326782	LOC105320676			
LOC105320778	LOC105322883	LOC105328743			
LOC105344843	LOC105345733	LOC105341715			
LOC105325734	LOC105322140	LOC105326502			
LOC105334974	LOC105338572	LOC105341308			
LOC105335278	LOC105339136	LOC105331606			
LOC105346538	LOC105332743	LOC105322302			
LOC105322986	LOC105323677	LOC105335965			

LOC105336172	LOC105330881	LOC105332517
LOC105329738	LOC105325879	LOC105326520
LOC105320145	LOC105330789	LOC105346629
LOC105319300	LOC105320756	LOC105336047
LOC105320307	LOC105335065	LOC105344085
LOC105340013	LOC105324402	LOC105326650
LOC105323677	LOC105346745	LOC105318265
LOC105339609	LOC105326445	LOC105329353
LOC105347073	LOC105320234	LOC105317941
LOC105335482	LOC105337688	LOC105333374
LOC105329230	LOC105333532	LOC105329676
LOC105328901	LOC105347939	LOC105330238
LOC105318574	LOC105334786	LOC105319362
LOC105338924	LOC105320494	LOC105328612
LOC105348177	LOC105340890	LOC105323666
LOC105321958	LOC105337906	LOC105334215
LOC105322883	LOC105318519	LOC105318191
LOC105322211	LOC105349083	LOC105346887
LOC105329328	LOC105320531	LOC105343744
LOC105348718	LOC105336045	LOC105337641
LOC105339801	LOC105334049	LOC105344054
LOC105330815	LOC105319692	LOC105329418
LOC105327565	LOC105342011	LOC105346527
LOC105332173	LOC105344724	LOC105319189
LOC105346405	LOC105346253	LOC105344015
LOC105345529	LOC105336665	LOC105336364
LOC105338351	LOC105324151	LOC105336253
LOC105333077	LOC105332966	LOC105344924
LOC105345273	LOC105339019	LOC105335061
LOC105340864	LOC105318220	LOC105330797
LOC105342681	LOC105321955	LOC105322992
LOC105348052	LOC105327971	LOC105331147
LOC105344149	LOC105338229	LOC105347967
LOC105337238	LOC105344149	LOC105342681
LOC105338434	LOC105343676	LOC105342423

LOC105334815	LOC105327706	LOC105319632
LOC105348731	LOC105332019	LOC105324743
LOC105343393	LOC105319484	LOC105330673
LOC105335965	LOC105323785	LOC105329684
LOC105331602	LOC105321551	LOC105344552
LOC105330173	LOC105335624	LOC105324078
LOC105322148	LOC105346536	LOC105344413
LOC105335829	LOC105335118	LOC105348500
LOC105325625	LOC105331147	LOC105348059
LOC105343024	LOC105318087	LOC105331825
LOC105332254	LOC105328030	LOC105328358
LOC105324831	LOC105343596	LOC105319479
LOC105326391	LOC105331979	LOC105348287
LOC105318724	LOC105344914	LOC105343024
LOC105323026	LOC105317819	LOC105347142
LOC105330526	LOC105338275	LOC105322210
LOC105337422	LOC105326795	LOC105346416
LOC105328434	LOC105324897	LOC105322986
LOC105323674	LOC105346832	LOC105329817
LOC105342984	LOC105321691	LOC105340357
LOC105323237	LOC105329410	LOC105335309
LOC105334794	LOC105323590	LOC105333390
LOC105326101	LOC105331606	LOC105329230
LOC105332047	LOC105325189	LOC105322211
LOC105319059	LOC105330498	LOC105346534
LOC105318554	LOC105322572	LOC105321642
LOC105343026	LOC105337238	LOC105347695
LOC105331509	LOC105343695	LOC105336046
LOC105327534	LOC105327168	LOC105333647
LOC105317711	LOC105324777	LOC105324719
LOC105348350	LOC105332819	LOC105346764
LOC105344031	LOC105332880	LOC105344122
LOC105344040	LOC105334642	LOC105335272
LOC105327481	LOC105336047	LOC105338229
LOC105318847	LOC105342355	LOC105327104

LOC105346629	LOC105330607	LOC105321872
LOC105344842	LOC105330830	LOC105323732
LOC105325416	LOC105348350	LOC105324632
LOC105334142	LOC105329601	LOC105332047
LOC105338195	LOC105326683	LOC105318972
LOC105326609	LOC105322048	LOC105323785
LOC105326138	LOC105336663	LOC105326635
LOC105323605	LOC105332888	LOC105341948
LOC105323786	LOC105317259	LOC105348182
LOC105349162	LOC105344031	LOC105348656
LOC105322266	LOC105337459	LOC105345373
LOC105322691	LOC105330523	LOC105337186
LOC105336869	LOC105331968	LOC105336627
LOC105341308	LOC105335837	LOC105348493
LOC105333550	LOC105336869	LOC105318724
LOC105346047	LOC105324630	LOC105324777
LOC105320730	LOC105328891	LOC105347371
LOC105327205	LOC105324175	LOC105333787
LOC105322048	LOC105324352	LOC105331626
LOC105324738	LOC105349002	LOC105317600
LOC105324175	LOC105341308	LOC105338434
LOC105335413	LOC105324842	LOC105346832
LOC105337718	LOC105339524	LOC105317620
LOC105348973	LOC105330390	LOC105342419
LOC105348500	LOC105319578	LOC105330318
LOC105319632	LOC105334651	LOC105346777
LOC105329684	LOC105318191	LOC105329401
LOC105322767	LOC105339960	LOC105342079
LOC105344413	LOC105327897	LOC105325289
LOC105340128	LOC105348023	LOC105322984
LOC105324078	LOC105340159	LOC105322982
LOC105329557	LOC105342192	LOC105340527
LOC105332529	LOC105320319	LOC105317508
LOC105339145	LOC105331451	LOC105322358
LOC105326787	LOC105341116	LOC105322985

LOC105327707	LOC105321535	LOC105334651
LOC105345521	LOC105346882	LOC105334722
LOC105321686	LOC105346605	LOC105331165
LOC105340050	LOC105320524	LOC105317819
LOC105320128	LOC105338421	LOC105325768
LOC105342527	LOC105328789	LOC105332939
LOC105324766	LOC105333640	LOC105333582
LOC105343463	LOC105319449	LOC105342749
LOC105318972	LOC105327036	LOC105338718
LOC105329541	LOC105326758	LOC105325510
LOC105348403	LOC105335032	LOC105322667
LOC105340359	LOC105341306	LOC105343026
LOC105322406	LOC105335236	LOC105317472
LOC105334652	LOC105322358	LOC105322983
LOC105346239	LOC105329193	LOC105318891
LOC105332779	LOC105322231	LOC105337688
LOC105346832	LOC105335743	LOC105347939
LOC105326311	LOC105327837	LOC105329492
LOC105326853	LOC105334792	LOC105335791
LOC105339524	LOC105338428	LOC105328916
LOC105322597	LOC105332542	LOC105322369
LOC105341155	LOC105343744	LOC105322705
LOC105344724	LOC105340984	LOC105334552
LOC105336952	LOC105334721	LOC105344695
LOC105346253	LOC105328379	LOC105346915
LOC105342512	LOC105328341	LOC105339434
LOC105326597	LOC105330238	LOC105334792
LOC105328316	LOC105329418	LOC105348139
LOC105323466	LOC105333462	LOC105317615
LOC105346685	LOC105341840	LOC105320711
LOC105342016	LOC105319189	LOC105331509
LOC105346232	LOC105342445	LOC105333357
LOC105346928	LOC105318243	LOC105322617
LOC105348436	LOC105329071	LOC105338448
LOC105343612	LOC105320548	LOC105341186

LOC105335351	LOC105336604	LOC105328391
LOC105334082	LOC105324737	LOC105330390
LOC105326445	LOC105330033	LOC105334246
LOC105346796	LOC105324693	LOC105333218
LOC105328824	LOC105340738	LOC105323674
LOC105346448	LOC105345719	LOC105347615
LOC105346745	LOC105337836	LOC105319380
LOC105320234	LOC105343776	LOC105331146
LOC105331084	LOC105322911	LOC105318799
LOC105329509	LOC105320504	LOC105319670
LOC105339358	LOC105324055	LOC105323026
LOC105326707	LOC105337533	LOC105334794
LOC105331913	LOC105333582	LOC105334946
LOC105327279	LOC105331165	LOC105331368
LOC105335370	LOC105327279	LOC105326391
LOC105341159	LOC105337625	LOC105347561
LOC105322985	LOC105323667	LOC105345439
LOC105326589	LOC105318833	LOC105327121
LOC105333582	LOC105325768	LOC105344121
LOC105345719	LOC105346777	LOC105338877
LOC105332312	LOC105326635	LOC105328693
LOC105322983	LOC105338718	LOC105326445
LOC105328747	LOC105325709	LOC105347683
LOC105338871	LOC105331865	LOC105344061
LOC105317622	LOC105325787	LOC105332341
LOC105319458	LOC105322641	LOC105333269
LOC105328771	LOC105337560	LOC105346745
LOC105325250	LOC105328968	
LOC105320111	LOC105319479	
LOC105336346	LOC105343024	
LOC105318604	LOC105336490	
LOC105344578	LOC105320128	
LOC105327533	LOC105341286	
LOC105328951	LOC105345824	
LOC105342482	LOC105333229	

LOC105348974	LOC105324766
LOC105348359	
LOC105338877	
LOC105348149	
LOC105327121	
LOC105331331	
LOC105340635	
LOC105343784	
LOC105322369	
LOC105347968	

Supplementary Table 4 The differentially expressed miRNAs and their targets in WS vs NS comparison

sRNA	WSM	NSM	log2.Fold_	p.value	q.value.	transcript_id	gene_id	length	WSM_FPK	NSM_FPKI	log2(foldcha	pvalue	qvalue
novel_9	23871.944	9601.8749	1.3139	0	0	XM_011418520.1	LOC105320548	975	0.197935	16.4551	-6.37737	7.97E-07	0.0003515
novel_9	23871.944	9601.8749	1.3139	0	0	XM_011436703.1	LOC105333636	2101	0.665061	15.8191	-4.57204	5.28E-08	3.46E-05
novel_9	23871.944	9601.8749	1.3139	0	0	XM_011431580.1	LOC105330033	1022	1.05702	19.3213	-4.19211	6.86E-07	0.0003087
novel_9	23871.944	9601.8749	1.3139	0	0	XM_011420241.1	LOC105321793	2025	0.432902	4.81591	-3.4757	8.03E-05	0.01297
novel_9	23871.944	9601.8749	1.3139	0	0	XM_011441145.1	LOC105336711	2646	0.706123	9.15996	-3.69735	2.67E-05	0.0057056
novel_9	23871.944	9601.8749	1.3139	0	0	XM_011453636.1	LOC105345500	814	0.346691	9.48121	-4.77335	9.59E-06	0.0025389
novel_9	23871.944	9601.8749	1.3139	0	0	XM_011421010.1	LOC105322358	894	1.90839	59.7958	-4.96961	1.42E-09	1.55E-06
novel_9	23871.944	9601.8749	1.3139	0	0	XM_011452894.1	LOC105344958	1991	0.364423	5.06114	-3.79578	0.0001028	0.0155501
novel_9	23871.944	9601.8749	1.3139	0	0	XM_011416126.1	LOC105318835	1676	0.358464	3.68636	-3.3623	0.0003802	0.0405849
novel_9	23871.944	9601.8749	1.3139	0	0	XM_011423906.1	LOC105324744	864	2.02183	24.9541	-3.62554	1.01E-05	0.0026237
novel_9	23871.944	9601.8749	1.3139	0	0	XM_011423838.1	LOC105324693	538	7.18374	71.2601	-3.31029	1.68E-05	0.0039565
novel_9	23871.944	9601.8749	1.3139	0	0	XM_011422733.1	LOC105323666	2278	0.0140485	3.64256	-8.01839	1.74E-07	9.58E-05
novel_9	23871.944	9601.8749	1.3139	0	0	XM_011423413.1	LOC105324352	697	198.685	622.826	-1.64835	0.0002853	0.0330056
novel_9	23871.944	9601.8749	1.3139	0	0	XM_011432711.1	LOC105330797	1356	32.9676	273.077	-3.05019	2.44E-10	3.39E-07
lgi-miR-317	4337.8954	1397.9883	1.6336	0	0	XM_011454999.1	LOC105346446	2005	0.152985	4.48564	-4.87385	4.28E-05	0.0082587
lgi-miR-317	4337.8954	1397.9883	1.6336	0	0	XM_011420586.1	LOC105322065	8176	0.712199	7.6634	-3.42763	7.48E-08	4.71E-05
lgi-miR-317	4337.8954	1397.9883	1.6336	0	0	XM_011432711.1	LOC105330797	1356	32.9676	273.077	-3.05019	2.44E-10	3.39E-07
lgi-miR-317	4337.8954	1397.9883	1.6336	0	0	XM_011416776.1	LOC105319299	8337	0.0787423	4.89242	-5.95727	1.61E-10	2.41E-07
lgi-miR-317	4337.8954	1397.9883	1.6336	0	0	XM_011418531.1	LOC105320531	451	8.73999	316.295	-5.17749	4.44E-12	1.09E-08
lgi-miR-317	4337.8954	1397.9883	1.6336	0	0	XM_011416604.1	LOC105319189	3028	0.0096713	3.18469	-8.36322	0.0001752	0.022969
lgi-miR-317	4337.8954	1397.9883	1.6336	0	0	XM_011447258.1	LOC105340984	3799	0.233573	2.17515	-3.21917	0.00027	0.0316513
lgi-miR-317	4337.8954	1397.9883	1.6336	0	0	XM_011444402.1	LOC105339019	974	1.39759	21.1681	-3.92088	2.39E-06	0.0008524
lgi-miR-317	4337.8954	1397.9883	1.6336	0	0	XM_011435866.1	LOC105333064	699	3.3363	69.004	-4.37036	2.23E-08	1.68E-05
lgi-miR-317	4337.8954	1397.9883	1.6336	0	0	XM_011439813.1	LOC105335743	2363	0.142488	5.10054	-5.16174	7.99E-06	0.0022181
lgi-miR-317	4337.8954	1397.9883	1.6336	0	0	XM_011441278.1	LOC105336820	2414	0.487364	6.86182	-3.81552	4.55E-06	0.0014357
lgi-miR-317	4337.8954	1397.9883	1.6336	0	0	XM_011415361.1	LOC105318316	4878	0.358602	2.6413	-2.88079	0.0004392	0.0451343
lgi-miR-317	4337.8954	1397.9883	1.6336	0	0	XM_011428186.1	LOC105327613	6798	0.416294	4.21544	-3.34001	1.52E-05	0.0036515
lgi-miR-317	4337.8954	1397.9883	1.6336	0	0	XM_011419968.1	LOC105321610	4635	0.160619	3.15682	-4.29676	6.15E-06	0.0018223
lgi-miR-317	4337.8954	1397.9883	1.6336	0	0	XM_011436453.1	LOC105333462	1524	41.6518	143.978	-1.7894	0.0001564	0.0211912
lgi-miR-317	4337.8954	1397.9883	1.6336	0	0	XM_011422733.1	LOC105323666	2278	0.0140485	3.64256	-8.01839	1.74E-07	9.58E-05
lgi-miR-317	4337.8954	1397.9883	1.6336	0	0	XM_011430565.1	LOC105329353	1190	3.36469	22.5224	-2.74282	0.0001718	0.0226991
lgi-miR-317	4337.8954	1397.9883	1.6336	0	0	XM_011439676.1	LOC105335667	5210	0.0550919	1.1623	-4.39901	0.0004875	0.0486209
lgi-miR-317	4337.8954	1397.9883	1.6336	0	0	XM_011431843.1	LOC105330238	3925	0.069366	2.96152	-5.41597	1.44E-05	0.0035146
lgi-miR-317	4337.8954	1397.9883	1.6336	0	0	XM_011458302.1	LOC105348744	2934	0.111701	3.14498	-4.81534	4.65E-05	0.0087471
lgi-miR-317	4337.8954	1397.9883	1.6336	0	0	XM_011432698.1	LOC105330790	2103	0.323472	5.68383	-4.13515	2.88E-05	0.0060579
lgi-miR-317	4337.8954	1397.9883	1.6336	0	0	XM_011441145.1	LOC105336711	2646	0.706123	9.15996	-3.69735	2.67E-05	0.0057056
lgi-miR-317	4337.8954	1397.9883	1.6336	0	0	XM_011452919.1	LOC105344960	5235	1.1421	6.88937	-2.59268	0.000122	0.0176452
lgi-miR-317	4337.8954	1397.9883	1.6336	0	0	XM_011419680.1	LOC105321418	941	0.203134	4.3115	-4.40769	0.0001952	0.0249751
lgi-miR-317	4337.8954	1397.9883	1.6336	0	0	XM_011423866.1	LOC105324712	2916	58.3064	175.476	-1.58955	0.0003318	0.0367722

lgi-miR-124	112.61465	15.531046	2.8582	4.66E-22	4.51E-22	XM_011451354.1	LOC105343857	500	1.94615	28.2428	-3.85919	3.12E-05	0.006477
lgi-miR-124	112.61465	15.531046	2.8582	4.66E-22	4.51E-22	XM_011435866.1	LOC105333064	699	3.3363	69.004	-4.37036	2.23E-08	1.68E-05
lgi-miR-124	112.61465	15.531046	2.8582	4.66E-22	4.51E-22	XM_011447160.1	LOC105340900	1242	0.427374	4.56386	-3.41668	0.0004522	0.0460656
novel_203	32643.024	12008.41	1.4427	0	0	XM_011444272.1	LOC105338945	608	0.313699	14.901	-5.56988	3.35E-06	0.0011177
novel_203	32643.024	12008.41	1.4427	0	0	XM_011429957.1	LOC105328916	1375	0	25.8586	#NAME?	0.0001662	0.0220848
novel_203	32643.024	12008.41	1.4427	0	0	XM_011439599.1	LOC105335624	417	0.156992	12.9015	-6.3607	6.19E-05	0.0107387
novel_203	32643.024	12008.41	1.4427	0	0	XM_011439607.1	LOC105335630	339	6.51138	171.565	-4.71964	2.97E-08	2.14E-05
lgi-miR-198	10351.623	5159.4134	1.0046	0	0	XM_011415769.1	LOC105318571	1983	0.875812	8.75418	-3.32128	7.57E-05	0.012398
lgi-miR-198	10351.623	5159.4134	1.0046	0	0	XM_011444674.1	LOC105339221	1368	0.810401	12.6642	-3.96598	2.50E-06	0.0008835
lgi-miR-198	10351.623	5159.4134	1.0046	0	0	XM_011428034.1	LOC105327510	1412	0.313148	12.3748	-5.30442	1.27E-06	0.0005115
lgi-miR-198	10351.623	5159.4134	1.0046	0	0	XM_011432698.1	LOC105330790	2103	0.323472	5.68383	-4.13515	2.88E-05	0.0060579
lgi-miR-198	10351.623	5159.4134	1.0046	0	0	XM_011435605.1	LOC105332880	1217	0	22.1298	#NAME?	0.0003782	0.0404388
lgi-miR-198	10351.623	5159.4134	1.0046	0	0	XM_011439131.1	LOC105335309	1298	0.647799	10.9166	-4.07483	2.98E-06	0.0010153
lgi-miR-198	10351.623	5159.4134	1.0046	0	0	XM_011435430.1	LOC105332743	855	0.740092	12.5491	-4.08373	1.21E-05	0.0030387
lgi-miR-198	10351.623	5159.4134	1.0046	0	0	XM_011455326.1	LOC105346652	2322	0.0225933	2.04184	-6.49783	0.0004893	0.0487603
lgi-miR-198	10351.623	5159.4134	1.0046	0	0	XM_011446580.1	LOC105340509	3227	0.0383747	2.0925	-5.76893	1.88E-06	0.0007095
lgi-miR-198	10351.623	5159.4134	1.0046	0	0	XM_011439396.1	LOC105335502	1692	0.568008	7.81936	-3.78307	4.98E-05	0.0092011
lgi-miR-198	10351.623	5159.4134	1.0046	0	0	XM_011451012.1	LOC105343596	929	1.77713	71.5809	-5.33196	9.05E-12	2.03E-08
lgi-miR-198	10351.623	5159.4134	1.0046	0	0	XM_011445179.1	LOC105339584	641	30.7783	179.151	-2.54119	6.77E-06	0.001955
lgi-miR-198	10351.623	5159.4134	1.0046	0	0	XM_011436188.1	LOC105333283	882	0.753569	8.19822	-3.4435	0.0003034	0.0345701
lgi-miR-198	10351.623	5159.4134	1.0046	0	0	XM_011442468.1	LOC105337639	3936	0.916521	5.59174	-2.60906	0.0003257	0.0362563
lgi-miR-198	10351.623	5159.4134	1.0046	0	0	XM_011434645.1	LOC105332180	845	308.293	1101.8	-1.83749	3.13E-05	0.0064837
lgi-miR-198	10351.623	5159.4134	1.0046	0	0	XM_011458768.1	LOC105349096	1715	0.135731	3.20349	-4.56082	2.75E-05	0.0058414
lgi-miR-198	10351.623	5159.4134	1.0046	0	0	XM_011425613.1	LOC105325879	1083	1.25169	11.4275	-3.19056	0.0001999	0.0253881
lgi-miR-198	10351.623	5159.4134	1.0046	0	0	XM_011435866.1	LOC105333064	699	3.3363	69.004	-4.37036	2.23E-08	1.68E-05
lgi-miR-198	10351.623	5159.4134	1.0046	0	0	XM_011449378.1	LOC105342435	2439	0.0269134	1.84131	-6.09626	5.95E-05	0.0104019
lgi-miR-198	10351.623	5159.4134	1.0046	0	0	XM_011434464.1	LOC105332036	616	0.225644	13.9202	-5.94699	5.31E-06	0.0016175
lgi-miR-198	10351.623	5159.4134	1.0046	0	0	XM_011429921.1	LOC105328891	6100	0	5.2965	#NAME?	0.0001246	0.0179071
lgi-miR-198	10351.623	5159.4134	1.0046	0	0	XM_011431363.1	LOC105329879	4174	0.369255	3.47337	-3.23365	8.19E-05	0.0131633
lgi-miR-198	10351.623	5159.4134	1.0046	0	0	XM_011415387.1	LOC105318336	2256	0.251508	2.7897	-3.47143	0.0002387	0.0288849
lgi-miR-198	10351.623	5159.4134	1.0046	0	0	XM_011427804.1	LOC105327372	3664	0.19254	2.61358	-3.7628	2.35E-05	0.0051585
lgi-miR-198	10351.623	5159.4134	1.0046	0	0	XM_011431847.1	LOC105330241	970	11.0052	58.8848	-2.41972	8.40E-05	0.0133881
lgi-miR-198	10351.623	5159.4134	1.0046	0	0	XM_011444542.1	LOC105339129	4020	0.778683	4.89721	-2.65285	0.0003752	0.0402053
lgi-miR-198	10351.623	5159.4134	1.0046	0	0	XM_011426970.1	LOC105326792	1953	11.1089	42.9263	-1.95015	0.0002733	0.03195
lgi-miR-198	10351.623	5159.4134	1.0046	0	0	XM_011442054.1	LOC105337364	3967	0.627634	7.93268	-3.65981	4.80E-06	0.0014932
novel_207	14.962786	0	4.9033	3.65E-05	2.86E-05	XM_011439813.1	LOC105335743	2363	0.142488	5.10054	-5.16174	7.99E-06	0.0022181
novel_207	14.962786	0	4.9033	3.65E-05	2.86E-05	XM_011454952.1	LOC105346405	1362	0.0763793	4.62405	-5.91983	5.90E-06	0.0017615
novel_207	14.962786	0	4.9033	3.65E-05	2.86E-05	XM_011453711.1	LOC105345529	1592	0.179496	3.73512	-4.37913	0.000158	0.0213285
novel_207	14.962786	0	4.9033	3.65E-05	2.86E-05	XM_011443415.1	LOC105338351	3410	0.505475	6.12537	-3.59908	4.64E-05	0.0087324
novel_207	14.962786	0	4.9033	3.65E-05	2.86E-05	XM_011453377.1	LOC105345273	1416	0.0242963	2.68921	-6.7903	1.59E-05	0.003797
novel_207	14.962786	0	4.9033	3.65E-05	2.86E-05	XM_011441878.1	LOC105337238	3725	0.0392317	4.00818	-6.67479	3.25E-06	0.0010892
novel_207	14.962786	0	4.9033	3.65E-05	2.86E-05	XM_011443563.1	LOC105338434	4142	0	7.31274	#NAME?	0.0001843	0.0238868
novel_207	14.962786	0	4.9033	3.65E-05	2.86E-05	XM_011429957.1	LOC105328916	1375	0	25.8586	#NAME?	0.0001662	0.0220848
novel_207	14.962786	0	4.9033	3.65E-05	2.86E-05	XM_011450749.1	LOC105343393	1138	0.53102	93.3498	-7.45774	6.00E-15	3.39E-11

novel_207	14.962786	0	4.9033	3.65E-05	2.86E-05	XM_011433869.1	LOC105331602	4893	0.281562	10.6084	-5.23561	1.85E-10	2.67E-07
novel_207	14.962786	0	4.9033	3.65E-05	2.86E-05	XM_011420699.1	LOC105322148	20694	0.190644	7.17184	-5.23339	3.04E-14	1.37E-10
novel_207	14.962786	0	4.9033	3.65E-05	2.86E-05	XM_011421010.1	LOC105322358	894	1.90839	59.7958	-4.96961	1.42E-09	1.55E-06
novel_207	14.962786	0	4.9033	3.65E-05	2.86E-05	XM_011425315.1	LOC105325625	13468	0.19384	1.60577	-3.05033	0.0001324	0.0187324
novel_76	47.775913	21.16105	1.1749	0.0001686	0.00012781	XM_011450181.1	LOC105343024	740	6.30198	454.434	-6.17212	0	0
novel_76	47.775913	21.16105	1.1749	0.0001686	0.00012781	XM_011415387.1	LOC105318336	2256	0.251508	2.7897	-3.47143	0.0002387	0.0288849
novel_76	47.775913	21.16105	1.1749	0.0001686	0.00012781	XM_011444542.1	LOC105339129	4020	0.778683	4.89721	-2.65285	0.0003752	0.0402053
lgi-miR-745a	741.05166	206.17463	1.8457	2.12E-86	3.86E-86	XM_011444272.1	LOC105338945	608	0.313699	14.901	-5.56988	3.35E-06	0.0011177
lgi-miR-745a	741.05166	206.17463	1.8457	2.12E-86	3.86E-86	XM_011452894.1	LOC105344958	1991	0.364423	5.06114	-3.79578	0.0001028	0.0155501
lgi-miR-745a	741.05166	206.17463	1.8457	2.12E-86	3.86E-86	XM_011451604.1	LOC105344031	637	0.42776	10.5814	-4.62859	4.19E-05	0.0081185
lgi-miR-745a	741.05166	206.17463	1.8457	2.12E-86	3.86E-86	XM_011436453.1	LOC105333462	1524	41.6518	143.978	-1.7894	0.0001564	0.0211912
lgi-miR-745a	741.05166	206.17463	1.8457	2.12E-86	3.86E-86	XM_011420241.1	LOC105321793	2025	0.432902	4.81591	-3.4757	8.03E-05	0.01297
lgi-miR-745a	741.05166	206.17463	1.8457	2.12E-86	3.86E-86	XM_011458597.1	LOC105348973	807	13.4355	72.8356	-2.43859	8.61E-05	0.0136415
lgi-miR-981	18.900361	2.5237949	2.9047	6.78E-05	5.23E-05	XM_011416126.1	LOC105318835	1676	0.358464	3.68636	-3.3623	0.0003802	0.0405849
lgi-miR-981	18.900361	2.5237949	2.9047	6.78E-05	5.23E-05	XM_011452894.1	LOC105344958	1991	0.364423	5.06114	-3.79578	0.0001028	0.0155501
lgi-miR-981	18.900361	2.5237949	2.9047	6.78E-05	5.23E-05	XM_011421010.1	LOC105322358	894	1.90839	59.7958	-4.96961	1.42E-09	1.55E-06
lgi-miR-981	18.900361	2.5237949	2.9047	6.78E-05	5.23E-05	XM_011422733.1	LOC105323666	2278	0.0140485	3.64256	-8.01839	1.74E-07	9.58E-05
lgi-miR-981	18.900361	2.5237949	2.9047	6.78E-05	5.23E-05	XM_011423413.1	LOC105324352	697	198.685	622.826	-1.64835	0.0002853	0.0330056
lgi-miR-981	18.900361	2.5237949	2.9047	6.78E-05	5.23E-05	XM_011432711.1	LOC105330797	1356	32.9676	273.077	-3.05019	2.44E-10	3.39E-07
lgi-miR-981	18.900361	2.5237949	2.9047	6.78E-05	5.23E-05	XM_011423838.1	LOC105324693	538	7.18374	71.2601	-3.31029	1.68E-05	0.0039565
lgi-miR-981	18.900361	2.5237949	2.9047	6.78E-05	5.23E-05	XM_011423906.1	LOC105324744	864	2.02183	24.9541	-3.62554	1.01E-05	0.0026237
lgi-miR-981	18.900361	2.5237949	2.9047	6.78E-05	5.23E-05	XM_011431580.1	LOC105330033	1022	1.05702	19.3213	-4.19211	6.86E-07	0.0003087
lgi-miR-981	18.900361	2.5237949	2.9047	6.78E-05	5.23E-05	XM_011436703.1	LOC105333636	2101	0.665061	15.8191	-4.57204	5.28E-08	3.46E-05
lgi-miR-981	18.900361	2.5237949	2.9047	6.78E-05	5.23E-05	XM_011418520.1	LOC105320548	975	0.197935	16.4551	-6.37737	7.97E-07	0.0003515
lgi-miR-981	18.900361	2.5237949	2.9047	6.78E-05	5.23E-05	XM_011441145.1	LOC105336711	2646	0.706123	9.15996	-3.69735	2.67E-05	0.0057056
lgi-miR-981	18.900361	2.5237949	2.9047	6.78E-05	5.23E-05	XM_011453636.1	LOC105345500	814	0.346691	9.48121	-4.77335	9.59E-06	0.0025389
lgi-miR-133-	28169.938	10273.981	1.4552	0	0	XM_011457967.1	LOC105348500	1775	0.484511	4.86021	-3.32642	0.0001935	0.0248465
lgi-miR-133-	28169.938	10273.981	1.4552	0	0	XM_011428034.1	LOC105327510	1412	0.313148	12.3748	-5.30442	1.27E-06	0.0005115
lgi-miR-133-	28169.938	10273.981	1.4552	0	0	XM_011431843.1	LOC105330238	3925	0.069366	2.96152	-5.41597	1.44E-05	0.0035146
lgi-miR-133-	28169.938	10273.981	1.4552	0	0	XM_011418531.1	LOC105320531	451	8.73999	316.295	-5.17749	4.44E-12	1.09E-08
lgi-miR-133-	28169.938	10273.981	1.4552	0	0	XM_011431025.1	LOC105329684	433	3.46539	85.7733	-4.62944	5.76E-08	3.73E-05
lgi-miR-133-	28169.938	10273.981	1.4552	0	0	XM_011454999.1	LOC105346446	2005	0.152985	4.48564	-4.87385	4.28E-05	0.0082587
lgi-miR-133-	28169.938	10273.981	1.4552	0	0	XM_011452230.1	LOC105344413	2684	0.771609	16.3564	-4.40584	6.81E-09	5.99E-06
lgi-miR-133-	28169.938	10273.981	1.4552	0	0	XM_011423133.1	LOC105324078	3824	0	8.51582	#NAME?	0.0001281	0.0182798
novel_263	12.862746	2.717933	2.2426	0.0037786	0.0026854	XM_011430842.1	LOC105329557	1754	0.491206	7.39153	-3.91147	6.51E-06	0.0018973
novel_263	12.862746	2.717933	2.2426	0.0037786	0.0026854	XM_011435156.1	LOC105332529	2392	0.372576	3.76309	-3.33631	0.0001566	0.0212057
novel_263	12.862746	2.717933	2.2426	0.0037786	0.0026854	XM_011426963.1	LOC105326787	1140	0.285303	4.14685	-3.86145	0.0002541	0.0302387
novel_263	12.862746	2.717933	2.2426	0.0037786	0.0026854	XM_011428315.1	LOC105327707	1130	0.160271	13.7387	-6.42159	6.04E-09	5.40E-06
novel_263	12.862746	2.717933	2.2426	0.0037786	0.0026854	XM_011432264.1	LOC105330526	1542	0.065866	2.12856	-5.0142	0.0001869	0.0241558
novel_263	12.862746	2.717933	2.2426	0.0037786	0.0026854	XM_011426970.1	LOC105326792	1953	11.1089	42.9263	-1.95015	0.0002733	0.03195
novel_263	12.862746	2.717933	2.2426	0.0037786	0.0026854	XM_011453672.1	LOC105345521	24663	0.966006	3.68449	-1.93136	0.0002782	0.03236
novel_263	12.862746	2.717933	2.2426	0.0037786	0.0026854	XM_011415361.1	LOC105318316	4878	0.358602	2.6413	-2.88079	0.0004392	0.0451343
lgi-miR-34	284.81794	60.765217	2.2287	5.03E-42	6.02E-42	XM_011417933.1	LOC105320128	585	12.6065	73.8088	-2.54962	0.0002185	0.0270738
lgi-miR-34	284.81794	60.765217	2.2287	5.03E-42	6.02E-42	XM_011416776.1	LOC105319299	8337	0.0787423	4.89242	-5.95727	1.61E-10	2.41E-07

lgi-miR-34	284.81794	60.765217	2.2287	5.03E-42	6.02E-42	XM_011439725.1	LOC105335690	1166	0.832024	33.624	-5.33672	7.52E-11	1.25E-07
lgi-miR-34	284.81794	60.765217	2.2287	5.03E-42	6.02E-42	XM_011423940.1	LOC105324766	3569	0.332081	3.61726	-3.44529	0.0001095	0.0163428
lgi-miR-34	284.81794	60.765217	2.2287	5.03E-42	6.02E-42	XM_011427804.1	LOC105327372	3664	0.19254	2.61358	-3.7628	2.35E-05	0.0051585
lgi-miR-34	284.81794	60.765217	2.2287	5.03E-42	6.02E-42	XM_011429921.1	LOC105328891	6100	0	5.2965	#NAME?	0.0001246	0.0179071
lgi-miR-34	284.81794	60.765217	2.2287	5.03E-42	6.02E-42	XM_011428186.1	LOC105327613	6798	0.416294	4.21544	-3.34001	1.52E-05	0.0036515
lgi-let-7	26629.034	13123.734	1.0208	0	0	XM_011416312.1	LOC105318972	1840	0.0178807	65.2101	-11.8325	1.33E-15	8.33E-12
lgi-let-7	26629.034	13123.734	1.0208	0	0	XM_011423413.1	LOC105324352	697	198.685	622.826	-1.64835	0.0002853	0.0330056
lgi-let-7	26629.034	13123.734	1.0208	0	0	XM_011430825.1	LOC105329541	2201	0.0583943	3.11548	-5.73748	2.19E-06	0.0007957
novel_83	85.31413	7.1831087	3.5701	4.89E-20	4.54E-20	XM_011452919.1	LOC105344960	5235	1.1421	6.88937	-2.59268	0.000122	0.0176452
novel_83	85.31413	7.1831087	3.5701	4.89E-20	4.54E-20	XM_011449378.1	LOC105342435	2439	0.0269134	1.84131	-6.09626	5.95E-05	0.0104019
novel_83	85.31413	7.1831087	3.5701	4.89E-20	4.54E-20	XM_011446375.1	LOC105340359	1494	0.0683758	1.81342	-4.72909	0.0004694	0.0472288
novel_83	85.31413	7.1831087	3.5701	4.89E-20	4.54E-20	XM_011421102.1	LOC105322406	1200	0.0891922	2.55455	-4.84001	0.0003314	0.0367374
novel_83	85.31413	7.1831087	3.5701	4.89E-20	4.54E-20	XM_011431098.1	LOC105329738	1085	0.134999	3.91231	-4.857	9.99E-05	0.0152287
novel_83	85.31413	7.1831087	3.5701	4.89E-20	4.54E-20	XM_011423906.1	LOC105324744	864	2.02183	24.9541	-3.62554	1.01E-05	0.0026237
novel_83	85.31413	7.1831087	3.5701	4.89E-20	4.54E-20	XM_011449722.1	LOC105342697	3292	3.36	16.3299	-2.28098	0.0001222	0.0176459
novel_83	85.31413	7.1831087	3.5701	4.89E-20	4.54E-20	XM_011435485.1	LOC105332779	1498	0.340797	3.67179	-3.4295	0.0004259	0.0441238
novel_83	85.31413	7.1831087	3.5701	4.89E-20	4.54E-20	XM_011421972.1	LOC105323026	1097	0.632327	20.3242	-5.00639	9.21E-09	7.79E-06
novel_83	85.31413	7.1831087	3.5701	4.89E-20	4.54E-20	XM_011425315.1	LOC105325625	13468	0.19384	1.60577	-3.05033	0.0001324	0.0187324
novel_83	85.31413	7.1831087	3.5701	4.89E-20	4.54E-20	XM_011437475.1	LOC105334142	2027	0.672749	5.74478	-3.09411	0.0002639	0.0311912
novel_83	85.31413	7.1831087	3.5701	4.89E-20	4.54E-20	XM_011442468.1	LOC105337639	3936	0.916521	5.59174	-2.60906	0.0003257	0.0362563
lgi-miR-745l	4425.047	826.44577	2.4207	0	0	XM_011454952.1	LOC105346405	1362	0.0763793	4.62405	-5.91983	5.90E-06	0.0017615
lgi-miR-745l	4425.047	826.44577	2.4207	0	0	XM_011439607.1	LOC105335630	339	6.51138	171.565	-4.71964	2.97E-08	2.14E-05
lgi-miR-745l	4425.047	826.44577	2.4207	0	0	XM_011418531.1	LOC105320531	451	8.73999	316.295	-5.17749	4.44E-12	1.09E-08
lgi-miR-745l	4425.047	826.44577	2.4207	0	0	XM_011432698.1	LOC105330790	2103	0.323472	5.68383	-4.13515	2.88E-05	0.0060579
lgi-miR-745l	4425.047	826.44577	2.4207	0	0	XM_011434645.1	LOC105332180	845	308.293	1101.8	-1.83749	3.13E-05	0.0064837
lgi-miR-745l	4425.047	826.44577	2.4207	0	0	XM_011454756.1	LOC105346253	1913	9.00766	50.0368	-2.47376	6.16E-06	0.0018223
lgi-miR-281-	1273.6743	339.74163	1.9065	9.72E-153	2.21E-152	XM_011426731.1	LOC105326597	5753	0.0260125	0.508804	-4.28984	0.0003044	0.0346652
lgi-miR-281-	1273.6743	339.74163	1.9065	9.72E-153	2.21E-152	XM_011430842.1	LOC105329557	1754	0.491206	7.39153	-3.91147	6.51E-06	0.0018973
lgi-miR-281-	1273.6743	339.74163	1.9065	9.72E-153	2.21E-152	XM_011437475.1	LOC105334142	2027	0.672749	5.74478	-3.09411	0.0002639	0.0311912
lgi-miR-281-	1273.6743	339.74163	1.9065	9.72E-153	2.21E-152	XM_011439396.1	LOC105335502	1692	0.568008	7.81936	-3.78307	4.98E-05	0.0092011
lgi-miR-281-	1273.6743	339.74163	1.9065	9.72E-153	2.21E-152	XM_011445214.1	LOC105339609	1994	0.587487	7.02084	-3.57901	2.23E-05	0.0049581
lgi-miR-281-	1273.6743	339.74163	1.9065	9.72E-153	2.21E-152	XM_011420699.1	LOC105322148	20694	0.190644	7.17184	-5.23339	3.04E-14	1.37E-10
lgi-miR-281-	1273.6743	339.74163	1.9065	9.72E-153	2.21E-152	XM_011444272.1	LOC105338945	608	0.313699	14.901	-5.56988	3.35E-06	0.0011177
novel_30	2941.8937	808.97335	1.8626	0	0	XM_011429120.1	LOC105328316	2651	0.605952	5.72002	-3.23874	7.85E-05	0.0127393
novel_30	2941.8937	808.97335	1.8626	0	0	XM_011415964.1	LOC105318724	3401	0.390753	4.99197	-3.67528	8.05E-06	0.0022276
novel_30	2941.8937	808.97335	1.8626	0	0	XM_011450749.1	LOC105343393	1138	0.53102	93.3498	-7.45774	6.00E-15	3.39E-11
novel_30	2941.8937	808.97335	1.8626	0	0	XM_011427984.1	LOC105327481	1967	0.47405	6.15482	-3.69861	7.15E-05	0.0118909
novel_30	2941.8937	808.97335	1.8626	0	0	XM_011443563.1	LOC105338434	4142	0	7.31274	#NAME?	0.0001843	0.0238868
novel_30	2941.8937	808.97335	1.8626	0	0	XM_011418531.1	LOC105320531	451	8.73999	316.295	-5.17749	4.44E-12	1.09E-08
novel_30	2941.8937	808.97335	1.8626	0	0	XM_011441878.1	LOC105337238	3725	0.0392317	4.00818	-6.67479	3.25E-06	0.0010892
novel_30	2941.8937	808.97335	1.8626	0	0	XM_011453377.1	LOC105345273	1416	0.0242963	2.68921	-6.7903	1.59E-05	0.003797
novel_30	2941.8937	808.97335	1.8626	0	0	XM_011454952.1	LOC105346405	1362	0.0763793	4.62405	-5.91983	5.90E-06	0.0017615
lgi-miR-193	594.57386	134.14941	2.148	1.78E-82	2.99E-82	XM_011434634.1	LOC105332173	2190	0.162064	2.37011	-3.87032	0.0001269	0.0181643
lgi-miR-193	594.57386	134.14941	2.148	1.78E-82	2.99E-82	XM_011426494.1	LOC105326445	1190	5.08284	32.8605	-2.69265	0.0001059	0.015937

lgi-miR-193	594.57386	134.14941	2.148	1.78E-82	2.99E-82	XM_011429853.1	LOC105328824	1591	0.52754	5.98955	-3.5051	0.0002594	0.0307342
lgi-miR-193	594.57386	134.14941	2.148	1.78E-82	2.99E-82	XM_011455456.1	LOC105346745	1881	0.34871	13.2701	-5.25001	9.01E-10	1.04E-06
lgi-miR-193	594.57386	134.14941	2.148	1.78E-82	2.99E-82	XM_011415964.1	LOC105318724	3401	0.390753	4.99197	-3.67528	8.05E-06	0.0022276
lgi-miR-193	594.57386	134.14941	2.148	1.78E-82	2.99E-82	XM_011433120.1	LOC105331084	11586	0.0799712	0.989202	-3.62871	9.68E-05	0.0148414
lgi-miR-193	594.57386	134.14941	2.148	1.78E-82	2.99E-82	XM_011439676.1	LOC105335667	5210	0.0550919	1.1623	-4.39901	0.0004875	0.0486209
lgi-miR-193	594.57386	134.14941	2.148	1.78E-82	2.99E-82	XM_011427984.1	LOC105327481	1967	0.47405	6.15482	-3.69861	7.15E-05	0.0118909
lgi-miR-2c	836.86599	215.10498	1.96	3.13E-104	6.48E-104	XM_011428186.1	LOC105327613	6798	0.416294	4.21544	-3.34001	1.52E-05	0.0036515
lgi-miR-2c	836.86599	215.10498	1.96	3.13E-104	6.48E-104	XM_011421010.1	LOC105322358	894	1.90839	59.7958	-4.96961	1.42E-09	1.55E-06
lgi-miR-2c	836.86599	215.10498	1.96	3.13E-104	6.48E-104	XM_011430789.1	LOC105329509	1230	0.473874	8.96274	-4.24137	5.29E-05	0.0096266
lgi-miR-2c	836.86599	215.10498	1.96	3.13E-104	6.48E-104	XM_011433869.1	LOC105331602	4893	0.281562	10.6084	-5.23561	1.85E-10	2.67E-07
lgi-miR-2c	836.86599	215.10498	1.96	3.13E-104	6.48E-104	XM_011444881.1	LOC105339358	2991	0.0516873	2.77274	-5.74536	4.95E-05	0.0091587
lgi-miR-2c	836.86599	215.10498	1.96	3.13E-104	6.48E-104	XM_011439396.1	LOC105335502	1692	0.568008	7.81936	-3.78307	4.98E-05	0.0092011
lgi-miR-2c	836.86599	215.10498	1.96	3.13E-104	6.48E-104	XM_011427679.1	LOC105327279	445	659.578	2038.9	-1.62818	0.0002512	0.0300089
lgi-miR-2c	836.86599	215.10498	1.96	3.13E-104	6.48E-104	XM_011430842.1	LOC105329557	1754	0.491206	7.39153	-3.91147	6.51E-06	0.0018973
lgi-miR-2c	836.86599	215.10498	1.96	3.13E-104	6.48E-104	XM_011418520.1	LOC105320548	975	0.197935	16.4551	-6.37737	7.97E-07	0.0003515
lgi-miR-2c	836.86599	215.10498	1.96	3.13E-104	6.48E-104	XM_011431843.1	LOC105330238	3925	0.069366	2.96152	-5.41597	1.44E-05	0.0035146
lgi-miR-2c	836.86599	215.10498	1.96	3.13E-104	6.48E-104	XM_011431580.1	LOC105330033	1022	1.05702	19.3213	-4.19211	6.86E-07	0.0003087
lgi-miR-2c	836.86599	215.10498	1.96	3.13E-104	6.48E-104	XM_011439198.1	LOC105335370	896	0.477287	32.2544	-6.0785	5.95E-11	1.04E-07
lgi-miR-2c	836.86599	215.10498	1.96	3.13E-104	6.48E-104	XM_011457727.1	LOC105348350	1076	0.0751287	29.1535	-8.60009	1.37E-06	0.0005451
lgi-miR-2c	836.86599	215.10498	1.96	3.13E-104	6.48E-104	XM_011437475.1	LOC105334142	2027	0.672749	5.74478	-3.09411	0.0002639	0.0311912
lgi-miR-2c	836.86599	215.10498	1.96	3.13E-104	6.48E-104	XM_011432264.1	LOC105330526	1542	0.065866	2.12856	-5.0142	0.0001869	0.0241558
lgi-miR-2c	836.86599	215.10498	1.96	3.13E-104	6.48E-104	XM_011429297.1	LOC105328434	2287	0.0139869	3.66124	-8.03211	1.65E-07	9.15E-05
lgi-miR-2c	836.86599	215.10498	1.96	3.13E-104	6.48E-104	XM_011429921.1	LOC105328891	6100	0	5.2965	#NAME?	0.0001246	0.0179071
lgi-miR-2c	836.86599	215.10498	1.96	3.13E-104	6.48E-104	XM_011416604.1	LOC105319189	3028	0.0096713	3.18469	-8.36322	0.0001752	0.022969
lgi-miR-2c	836.86599	215.10498	1.96	3.13E-104	6.48E-104	XM_011421901.1	LOC105322985	1116	14.9954	71.1319	-2.24598	6.88E-05	0.0115858
lgi-miR-2c	836.86599	215.10498	1.96	3.13E-104	6.48E-104	XM_011441878.1	LOC105337238	3725	0.0392317	4.00818	-6.67479	3.25E-06	0.0010892
lgi-miR-2c	836.86599	215.10498	1.96	3.13E-104	6.48E-104	XM_011439176.1	LOC105335351	722	0.11774	52.4154	-8.79824	4.30E-09	4.10E-06
lgi-miR-2c	836.86599	215.10498	1.96	3.13E-104	6.48E-104	XM_011436626.1	LOC105333582	787	5.29869	34.3379	-2.69609	0.0003011	0.0343511
lgi-miR-2c	836.86599	215.10498	1.96	3.13E-104	6.48E-104	XM_011453997.1	LOC105345719	2532	0	7.84104	#NAME?	0.0004134	0.043166
novel_71	265.39257	30.673815	3.113	2.59E-53	3.37E-53	XM_011421899.1	LOC105322983	1009	0.333547	4.85432	-3.86331	0.0002523	0.0301002
novel_71	265.39257	30.673815	3.113	2.59E-53	3.37E-53	XM_011416781.1	LOC105319300	743	0	55.5274	#NAME?	0.0001424	0.0197278
novel_71	265.39257	30.673815	3.113	2.59E-53	3.37E-53	XM_011435430.1	LOC105332743	855	0.740092	12.5491	-4.08373	1.21E-05	0.0030387
novel_71	265.39257	30.673815	3.113	2.59E-53	3.37E-53	XM_011434634.1	LOC105332173	2190	0.162064	2.37011	-3.87032	0.0001269	0.0181643
novel_71	265.39257	30.673815	3.113	2.59E-53	3.37E-53	XM_011431847.1	LOC105330241	970	11.0052	58.8848	-2.41972	8.40E-05	0.0133881
novel_71	265.39257	30.673815	3.113	2.59E-53	3.37E-53	XM_011427804.1	LOC105327372	3664	0.19254	2.61358	-3.7628	2.35E-05	0.0051585
novel_71	265.39257	30.673815	3.113	2.59E-53	3.37E-53	XM_011417003.1	LOC105319458	4714	0.0275508	1.17997	-5.42052	0.0004241	0.0440186
novel_71	265.39257	30.673815	3.113	2.59E-53	3.37E-53	XM_011457195.1	LOC105347939	682	4.36077	129.157	-4.8884	6.51E-11	1.12E-07
lgi-miR-198(	342.56904	111.43525	1.6202	3.34E-35	3.61E-35	XM_011455714.1	LOC105346928	4093	0.290658	2.56711	-3.14275	0.0004987	0.0494356
lgi-miR-198(	342.56904	111.43525	1.6202	3.34E-35	3.61E-35	XM_011429777.1	LOC105328771	888	0.834493	11.1954	-3.74587	4.71E-05	0.0088184
lgi-miR-198(	342.56904	111.43525	1.6202	3.34E-35	3.61E-35	XM_011422876.1	LOC105323803	1081	1.0851	37.1538	-5.09761	3.13E-10	4.16E-07
lgi-miR-198(	342.56904	111.43525	1.6202	3.34E-35	3.61E-35	XM_011415769.1	LOC105318571	1983	0.875812	8.75418	-3.32128	7.57E-05	0.012398
lgi-miR-198(	342.56904	111.43525	1.6202	3.34E-35	3.61E-35	XM_011417911.1	LOC105320111	4088	0.424664	6.18287	-3.86388	9.34E-07	0.0004016
lgi-miR-198(	342.56904	111.43525	1.6202	3.34E-35	3.61E-35	XM_011452367.1	LOC105344578	1073	3.52597	26.8851	-2.93071	6.94E-05	0.0116577
lgi-miR-87	82.426575	34.556577	1.2541	2.50E-07	2.10E-07	XM_011455326.1	LOC105346652	2322	0.0225933	2.04184	-6.49783	0.0004893	0.0487603

lgi-miR-87	82.426575	34.556577	1.2541	2.50E-07	2.10E-07	XM_011426001.1	LOC105326138	2062	0.0157088	6.2972	-8.64699	1.26E-08	1.02E-05
lgi-miR-87	82.426575	34.556577	1.2541	2.50E-07	2.10E-07	XM_011436188.1	LOC105333283	882	0.753569	8.19822	-3.4435	0.0003034	0.0345701
lgi-miR-87	82.426575	34.556577	1.2541	2.50E-07	2.10E-07	XM_011445214.1	LOC105339609	1994	0.587487	7.02084	-3.57901	2.23E-05	0.0049581
lgi-miR-87	82.426575	34.556577	1.2541	2.50E-07	2.10E-07	XM_011432264.1	LOC105330526	1542	0.065866	2.12856	-5.0142	0.0001869	0.0241558
lgi-miR-87	82.426575	34.556577	1.2541	2.50E-07	2.10E-07	XM_011442468.1	LOC105337639	3936	0.916521	5.59174	-2.60906	0.0003257	0.0362563
lgi-miR-87	82.426575	34.556577	1.2541	2.50E-07	2.10E-07	XM_011421029.1	LOC105322369	4968	0.0294942	6.30805	-7.74062	4.68E-08	3.12E-05
lgi-miR-87	82.426575	34.556577	1.2541	2.50E-07	2.10E-07	XM_011421102.1	LOC105322406	1200	0.0891922	2.55455	-4.84001	0.0003314	0.0367374
lgi-miR-87	82.426575	34.556577	1.2541	2.50E-07	2.10E-07	XM_011438370.1	LOC105334794	5800	0.0776545	8.56628	-6.78546	3.22E-11	6.01E-08
lgi-miR-87	82.426575	34.556577	1.2541	2.50E-07	2.10E-07	XM_011434464.1	LOC105332036	616	0.225644	13.9202	-5.94699	5.31E-06	0.0016175
lgi-miR-87	82.426575	34.556577	1.2541	2.50E-07	2.10E-07	XM_011431843.1	LOC105330238	3925	0.069366	2.96152	-5.41597	1.44E-05	0.0035146
lgi-miR-87	82.426575	34.556577	1.2541	2.50E-07	2.10E-07	XM_011418531.1	LOC105320531	451	8.73999	316.295	-5.17749	4.44E-12	1.09E-08
lgi-miR-87	82.426575	34.556577	1.2541	2.50E-07	2.10E-07	XM_011439676.1	LOC105335667	5210	0.0550919	1.1623	-4.39901	0.0004875	0.0486209
lgi-miR-200	795.91521	1770.5392	-1.1535	2.50E-60	3.67E-60	XM_011441845.1	LOC105337211	4415	1.63723	0.187215	3.12848	0.0004863	0.0485287
lgi-miR-200	795.91521	1770.5392	-1.1535	2.50E-60	3.67E-60	XM_011427152.1	LOC105326914	475	215.729	27.1654	2.98938	3.59E-06	0.001184
lgi-miR-200	795.91521	1770.5392	-1.1535	2.50E-60	3.67E-60	XM_011434384.1	LOC105331979	669	148.076	0.331872	8.8015	8.88E-15	4.65E-11
lgi-miR-200	795.91521	1770.5392	-1.1535	2.50E-60	3.67E-60	XM_011414199.1	LOC105317540	4097	1.94543	0.0498813	5.28545	9.03E-05	0.0141556
lgi-miR-200	795.91521	1770.5392	-1.1535	2.50E-60	3.67E-60	XM_011433278.1	LOC105331186	2065	64.6143	18.335	1.81725	0.0003079	0.0349134
novel_12	4029.1895	13601.702	-1.7552	0	0	XM_011415777.1	LOC105318575	934	15.9619	0.051236	8.28326	4.40E-08	2.97E-05
novel_12	4029.1895	13601.702	-1.7552	0	0	XM_011428249.1	LOC105327657	1807	3.50513	0.317393	3.46513	0.0002813	0.0326619
novel_12	4029.1895	13601.702	-1.7552	0	0	XM_011444553.1	LOC105339136	7269	8.17398	0.544605	3.90776	2.69E-07	0.0001386
novel_12	4029.1895	13601.702	-1.7552	0	0	XM_011452741.1	LOC105344843	658	8.94442	0.596098	3.90736	0.0003297	0.0366118
novel_12	4029.1895	13601.702	-1.7552	0	0	XM_011425426.1	LOC105325734	7535	6.47482	1.32013	2.29416	0.0002402	0.0290175
novel_12	4029.1895	13601.702	-1.7552	0	0	XM_011438626.1	LOC105334974	648	62.042	0.872494	6.15196	3.19E-11	6.00E-08
novel_12	4029.1895	13601.702	-1.7552	0	0	XM_011439097.1	LOC105335278	1014	15.6498	0.0459325	8.41241	2.56E-08	1.90E-05
novel_12	4029.1895	13601.702	-1.7552	0	0	XM_011455165.1	LOC105346538	1697	9440.96	1827.1	2.36938	4.43E-05	0.0084433
novel_12	4029.1895	13601.702	-1.7552	0	0	XM_011431656.1	LOC105330078	595	52.1328	1.01566	5.6817	1.36E-09	1.50E-06
novel_12	4029.1895	13601.702	-1.7552	0	0	XM_011440390.1	LOC105336172	1863	110.828	22.7855	2.28214	3.74E-06	0.0012234
novel_12	4029.1895	13601.702	-1.7552	0	0	XM_011418186.1	LOC105320307	2006	14.9621	0	inf	0.0001589	0.0214005
novel_12	4029.1895	13601.702	-1.7552	0	0	XM_011445843.1	LOC105340013	2428	1.2958	0.0729811	4.15018	0.0004689	0.047182
novel_12	4029.1895	13601.702	-1.7552	0	0	XM_011448772.1	LOC105341999	2179	3.72508	0.180433	4.36773	0.0003549	0.0386233
novel_12	4029.1895	13601.702	-1.7552	0	0	XM_011430063.1	LOC105328971	2146	7.98031	0	inf	0.0004585	0.0464445
novel_12	4029.1895	13601.702	-1.7552	0	0	XM_011437749.1	LOC105334348	5211	5.42431	0.85472	2.66592	0.0004267	0.0441814
novel_12	4029.1895	13601.702	-1.7552	0	0	XM_011430421.1	LOC105329230	7202	5.30808	0.705808	2.91084	2.76E-05	0.0058656
novel_12	4029.1895	13601.702	-1.7552	0	0	XM_011429938.1	LOC105328901	4819	2.62077	0.127041	4.36662	2.31E-05	0.0050871
novel_12	4029.1895	13601.702	-1.7552	0	0	XM_011415775.1	LOC105318574	1018	13.3346	0.137088	6.60393	2.20E-07	0.000117
novel_12	4029.1895	13601.702	-1.7552	0	0	XM_011457502.1	LOC105348177	4801	1.08137	0.0544965	4.31055	5.71E-05	0.0101311
novel_12	4029.1895	13601.702	-1.7552	0	0	XM_011420447.1	LOC105321958	4590	2.83367	0.182844	3.95399	4.49E-06	0.0014214
novel_12	4029.1895	13601.702	-1.7552	0	0	XM_011421785.1	LOC105322883	841	20.4909	2.84061	2.85071	0.00049	0.0488045
lgi-miR-184	48720.931	100372.88	-1.0428	0	0	XM_011458268.1	LOC105348718	10120	9.93626	1.7429	2.51121	8.07E-06	0.0022312
lgi-miR-184	48720.931	100372.88	-1.0428	0	0	XM_011445526.1	LOC105339801	3097	5.86212	0.479604	3.61151	3.31E-05	0.0067618
lgi-miR-184	48720.931	100372.88	-1.0428	0	0	XM_011428124.1	LOC105327565	1140	47.2652	1.3033	5.18054	6.91E-11	1.17E-07
lgi-miR-96b	7532.5814	18149.386	-1.2687	0	0	XM_011444248.1	LOC105338930	4143	6.02011	0.841484	2.83878	0.000243	0.0292635
lgi-miR-96b	7532.5814	18149.386	-1.2687	0	0	XM_011420678.1	LOC105322139	927	16.1232	1.60453	3.32892	8.28E-05	0.013259
lgi-miR-96b	7532.5814	18149.386	-1.2687	0	0	XM_011424032.1	LOC105324831	1859	287.63	88.131	1.70649	0.0001396	0.0194788

lgi-miR-96b	7532.5814	18149.386	-1.2687	0	0	XM_011416901.1	LOC105319380	3947	3.77713	0.138783	4.76638	7.65E-06	0.0021469
lgi-miR-96b	7532.5814	18149.386	-1.2687	0	0	XM_011442135.1	LOC105337422	1399	21.5943	0.223559	6.59385	2.40E-11	4.74E-08
lgi-miR-96b	7532.5814	18149.386	-1.2687	0	0	XM_011441029.1	LOC105336634	1719	10.5093	0.456396	4.52523	1.79E-07	9.81E-05
lgi-miR-96b	7532.5814	18149.386	-1.2687	0	0	XM_011425962.1	LOC105326101	1152	7.41477	0.077947	6.57176	1.74E-05	0.0040739
lgi-miR-96b	7532.5814	18149.386	-1.2687	0	0	XM_011434477.1	LOC105332047	2241	10.6197	0.142008	6.22463	1.41E-10	2.16E-07
lgi-miR-96b	7532.5814	18149.386	-1.2687	0	0	XM_011439093.1	LOC105335277	3336	4.45006	0.573712	2.95543	0.0003152	0.0354152
lgi-miR-96b	7532.5814	18149.386	-1.2687	0	0	XM_011450182.1	LOC105343026	656	374.519	84.2839	2.15171	1.52E-05	0.0036605
lgi-miR-96b	7532.5814	18149.386	-1.2687	0	0	XM_011428737.1	LOC105328017	1584	18.7833	0.608021	4.94918	2.62E-09	2.66E-06
lgi-miR-96b	7532.5814	18149.386	-1.2687	0	0	XM_011428085.1	LOC105327534	2090	8.16277	0.0348893	7.87013	1.77E-06	0.0006748
lgi-miR-96b	7532.5814	18149.386	-1.2687	0	0	XM_011414444.1	LOC105317711	2896	1.95315	0.135382	3.85069	0.0001401	0.0195188
lgi-miR-315	7539.4065	21664.644	-1.5228	0	0	XM_011418008.1	LOC105320181	2541	5.30573	0.468363	3.50185	0.0001248	0.0179229
lgi-miR-315	7539.4065	21664.644	-1.5228	0	0	XM_011457261.1	LOC105347962	3527	40.0247	0	inf	5.19E-06	0.001594
lgi-miR-315	7539.4065	21664.644	-1.5228	0	0	XM_011429938.1	LOC105328901	4819	2.62077	0.127041	4.36662	2.31E-05	0.0050871
lgi-miR-315	7539.4065	21664.644	-1.5228	0	0	XM_011445843.1	LOC105340013	2428	1.2958	0.0729811	4.15018	0.0004689	0.047182
lgi-miR-315	7539.4065	21664.644	-1.5228	0	0	XM_011441029.1	LOC105336634	1719	10.5093	0.456396	4.52523	1.79E-07	9.81E-05
lgi-miR-315	7539.4065	21664.644	-1.5228	0	0	XM_011451616.1	LOC105344040	2718	45.7333	9.66283	2.24273	1.70E-05	0.0040007
lgi-miR-315	7539.4065	21664.644	-1.5228	0	0	XM_011416141.1	LOC105318847	1804	2.54241	0.136286	4.22149	0.0001615	0.021603
lgi-miR-315	7539.4065	21664.644	-1.5228	0	0	XM_011452740.1	LOC105344842	681	14.9112	0.242115	5.94456	3.68E-06	0.0012096
lgi-miR-315	7539.4065	21664.644	-1.5228	0	0	XM_011418186.1	LOC105320307	2006	14.9621	0	inf	0.0001589	0.0214005
lgi-miR-315	7539.4065	21664.644	-1.5228	0	0	XM_011440390.1	LOC105336172	1863	110.828	22.7855	2.28214	3.74E-06	0.0012234
lgi-miR-315	7539.4065	21664.644	-1.5228	0	0	XM_011424966.1	LOC105325416	3046	31.5796	6.6197	2.25416	5.30E-05	0.0096355
lgi-miR-315	7539.4065	21664.644	-1.5228	0	0	XM_011425426.1	LOC105325734	7535	6.47482	1.32013	2.29416	0.0002402	0.0290175
lgi-miR-315	7539.4065	21664.644	-1.5228	0	0	XM_011448192.1	LOC105341599	2557	3.70164	0.167991	4.46171	3.89E-05	0.0076768
lgi-miR-315	7539.4065	21664.644	-1.5228	0	0	XM_011443196.1	LOC105338195	1138	13.9848	0.710726	4.29843	1.12E-05	0.0028529
lgi-miR-315	7539.4065	21664.644	-1.5228	0	0	XM_011426739.1	LOC105326609	539	843.77	224.979	1.90706	4.57E-05	0.0086326
lgi-miR-315	7539.4065	21664.644	-1.5228	0	0	XM_011452741.1	LOC105344843	658	8.94442	0.596098	3.90736	0.0003297	0.0366118
lgi-miR-315	7539.4065	21664.644	-1.5228	0	0	XM_011421785.1	LOC105322883	841	20.4909	2.84061	2.85071	0.00049	0.0488045
lgi-miR-315	7539.4065	21664.644	-1.5228	0	0	XM_011457502.1	LOC105348177	4801	1.08137	0.0544965	4.31055	5.71E-05	0.0101311
lgi-miR-315	7539.4065	21664.644	-1.5228	0	0	XM_011420447.1	LOC105321958	4590	2.83367	0.182844	3.95399	4.49E-06	0.0014214
lgi-miR-315	7539.4065	21664.644	-1.5228	0	0	XM_011427149.1	LOC105326913	1281	359.991	118.564	1.6023	0.0003772	0.040373
lgi-miR-315	7539.4065	21664.644	-1.5228	0	0	XM_011437749.1	LOC105334348	5211	5.42431	0.85472	2.66592	0.0004267	0.0441814
lgi-miR-315	7539.4065	21664.644	-1.5228	0	0	XM_011422797.1	LOC105323721	588	207.626	26.8123	2.95302	9.44E-07	0.0004047
lgi-miR-315	7539.4065	21664.644	-1.5228	0	0	XM_011415775.1	LOC105318574	1018	13.3346	0.137088	6.60393	2.20E-07	0.000117
lgi-miR-315	7539.4065	21664.644	-1.5228	0	0	XM_011439093.1	LOC105335277	3336	4.45006	0.573712	2.95543	0.0003152	0.0354152
lgi-miR-315	7539.4065	21664.644	-1.5228	0	0	XM_011422857.1	LOC105323786	652	12.7159	0.522721	4.60444	2.59E-05	0.0055949
lgi-miR-315	7539.4065	21664.644	-1.5228	0	0	XM_011448772.1	LOC105341999	2179	3.72508	0.180433	4.36773	0.0003549	0.0386233
lgi-miR-315	7539.4065	21664.644	-1.5228	0	0	XM_011420907.1	LOC105322266	3621	1.55762	0.0910781	4.09609	5.74E-05	0.0101708
lgi-miR-315	7539.4065	21664.644	-1.5228	0	0	XM_011421538.1	LOC105322691	2401	9.37901	0	inf	0.0001952	0.0249751
lgi-miR-315	7539.4065	21664.644	-1.5228	0	0	XM_011441344.1	LOC105336869	795	202.774	49.3527	2.03867	7.36E-05	0.0121418
lgi-miR-315	7539.4065	21664.644	-1.5228	0	0	XM_011414444.1	LOC105317711	2896	1.95315	0.135382	3.85069	0.0001401	0.0195188
lgi-miR-315	7539.4065	21664.644	-1.5228	0	0	XM_011454500.1	LOC105346047	767	14.8217	0.337536	5.45652	9.80E-07	0.0004166
lgi-miR-315	7539.4065	21664.644	-1.5228	0	0	XM_011455165.1	LOC105346538	1697	9440.96	1827.1	2.36938	4.43E-05	0.0084433
lgi-miR-315	7539.4065	21664.644	-1.5228	0	0	XM_011431656.1	LOC105330078	595	52.1328	1.01566	5.6817	1.36E-09	1.50E-06
lgi-miR-315	7539.4065	21664.644	-1.5228	0	0	XM_011423902.1	LOC105324738	2648	55.7163	15.3393	1.86087	0.0001815	0.0236316

lgi-miR-315	7539.4065	21664.644	-1.5228	0	0	XM_011438626.1	LOC105334974	648	62.042	0.872494	6.15196	3.19E-11	6.00E-08
lgi-miR-315	7539.4065	21664.644	-1.5228	0	0	XM_011440112.1	LOC105335965	1905	1.66695	0.0640068	4.70284	0.000406	0.0425867
lgi-miR-315	7539.4065	21664.644	-1.5228	0	0	XM_011423227.1	LOC105324175	483	75.7078	5.57554	3.76326	3.43E-06	0.0011399
lgi-miR-315	7539.4065	21664.644	-1.5228	0	0	XM_011415777.1	LOC105318575	934	15.9619	0.051236	8.28326	4.40E-08	2.97E-05
lgi-miR-315	7539.4065	21664.644	-1.5228	0	0	XM_011428249.1	LOC105327657	1807	3.50513	0.317393	3.46513	0.0002813	0.0326619
novel_8	283.76792	618.91217	-1.125	3.56E-21	3.38E-21	XM_011455552.1	LOC105346832	2765	50.5387	5.31867	3.24825	2.40E-08	1.79E-05
novel_8	283.76792	618.91217	-1.125	3.56E-21	3.38E-21	XM_011417231.1	LOC105319632	1627	3.74101	0.0256155	7.19027	3.32E-06	0.0011093
novel_8	283.76792	618.91217	-1.125	3.56E-21	3.38E-21	XM_011426266.1	LOC105326311	523	52.0024	5.6665	3.19805	8.12E-05	0.0130658
novel_8	283.76792	618.91217	-1.125	3.56E-21	3.38E-21	XM_011433228.1	LOC105331147	2007	2.33225	0.0804128	4.85815	7.00E-05	0.0117033
novel_8	283.76792	618.91217	-1.125	3.56E-21	3.38E-21	XM_011445101.1	LOC105339524	1733	16.4326	1.45631	3.49617	4.51E-05	0.0085552
novel_8	283.76792	618.91217	-1.125	3.56E-21	3.38E-21	XM_011421414.1	LOC105322597	425	19.255	0.771449	4.64152	0.0001585	0.0213622
novel_8	283.76792	618.91217	-1.125	3.56E-21	3.38E-21	XM_011428249.1	LOC105327657	1807	3.50513	0.317393	3.46513	0.0002813	0.0326619
lgi-miR-153	326.03123	1020.3897	-1.646	1.82E-66	2.85E-66	XM_011420084.1	LOC105321686	2992	10.1301	1.3163	2.94409	4.52E-05	0.0085599
lgi-miR-153	326.03123	1020.3897	-1.646	1.82E-66	2.85E-66	XM_011455385.1	LOC105346685	3130	1.48726	0.0860188	4.11186	0.0001399	0.0195007
lgi-miR-153	326.03123	1020.3897	-1.646	1.82E-66	2.85E-66	XM_011441029.1	LOC105336634	1719	10.5093	0.456396	4.52523	1.79E-07	9.81E-05
lgi-miR-153	326.03123	1020.3897	-1.646	1.82E-66	2.85E-66	XM_011448772.1	LOC105341999	2179	3.72508	0.180433	4.36773	0.0003549	0.0386233
lgi-miR-153	326.03123	1020.3897	-1.646	1.82E-66	2.85E-66	XM_011420907.1	LOC105322266	3621	1.55762	0.0910781	4.09609	5.74E-05	0.0101708
lgi-miR-153	326.03123	1020.3897	-1.646	1.82E-66	2.85E-66	XM_011421538.1	LOC105322691	2401	9.37901	0	inf	0.0001952	0.0249751
lgi-miR-153	326.03123	1020.3897	-1.646	1.82E-66	2.85E-66	XM_011428799.1	LOC105328052	1344	2.38029	0.0321885	6.20845	7.82E-05	0.0127039
lgi-miR-153	326.03123	1020.3897	-1.646	1.82E-66	2.85E-66	XM_011457834.1	LOC105348436	1321	5.54255	0.16551	5.06556	0.0003456	0.037862
lgi-miR-153	326.03123	1020.3897	-1.646	1.82E-66	2.85E-66	XM_011451032.1	LOC105343612	1744	11.9395	1.69661	2.81501	0.0002666	0.0313855
lgi-miR-153	326.03123	1020.3897	-1.646	1.82E-66	2.85E-66	XM_011450855.1	LOC105343463	298	1141.37	276.622	2.04477	0.0002035	0.0256922
lgi-miR-153	326.03123	1020.3897	-1.646	1.82E-66	2.85E-66	XM_011458268.1	LOC105348718	10120	9.93626	1.7429	2.51121	8.07E-06	0.0022312
lgi-miR-153	326.03123	1020.3897	-1.646	1.82E-66	2.85E-66	XM_011435888.1	LOC105333077	2809	2.49787	0.276491	3.1754	0.0004827	0.0482573
lgi-miR-190	1.5750301	15.531046	-3.3017	0.0006717	0.00048494	XM_011457739.1	LOC105348359	753	61.4677	7.40494	3.05327	6.95E-05	0.0116582
lgi-miR-190	1.5750301	15.531046	-3.3017	0.0006717	0.00048494	XM_011444163.1	LOC105338877	4827	13.4236	1.66008	3.01544	1.98E-06	0.0007386
lgi-miR-190	1.5750301	15.531046	-3.3017	0.0006717	0.00048494	XM_011413764.1	LOC105317200	1699	2.86189	0.170453	4.06952	0.000168	0.0222876
lgi-miR-190	1.5750301	15.531046	-3.3017	0.0006717	0.00048494	XM_011457470.1	LOC105348149	3906	31.7553	7.15637	2.1497	4.82E-05	0.0089693
lgi-miR-190	1.5750301	15.531046	-3.3017	0.0006717	0.00048494	XM_011431780.1	LOC105330173	1168	20.8175	0	inf	0.0002021	0.0255888
lgi-miR-190	1.5750301	15.531046	-3.3017	0.0006717	0.00048494	XM_011454916.1	LOC105346372	949	26.7948	0.200601	7.06148	1.03E-09	1.17E-06

Supplementary Table 5 The list of miRNAs targeted to mRNAs involving pigmentation

Gene_id/Gene description	Related pigments	Targeted microRNAs				
LOC105344040	melanin	**lgi-miR-2c**	lgi-miR-2d	**lgi-miR-315**	lgi-miR-71	**novel_60**
Tyrosinase-like protein 2		novel_211	novel_242	novel_248		
LOC105324831	melanin	lgi-miR-29	**lgi-miR-7**	**lgi-miR-96b**	novel_183	novel_24
Tyrosinase-like protein 3		novel_209	novel_220			
LOC105334556	melanin	**novel_12**	novel_73			
Dopamine beta-monooxygenase						
LOC105324712	melanin,	lgi-miR-1990	**lgi-miR-317**	novel_129	novel_134	novel_139
Chorion peroxidase	tetrapyrrole	novel_140	novel_157	**novel_188**	novel_272	
LOC105336634	carotenoid,melanin	**lgi-miR-153**	**lgi-miR-1985**	**lgi-miR-315**	lgi-miR-31	**lgi-miR-96b**
Cytochrome P450 2U1	tetrapyrrole,	novel_108	novel_227	novel_237	novel_24	
LOC105326901	melanin	lgi-miR-216b	lgi-miR-8	novel_100	novel_131	novel_134
Kynurenine 3- Monooxygenase	ommochrome,	novel_162				
